# The prevalence of gestational diabetes among underweight and normal weight women worldwide: a scoping review

**DOI:** 10.3389/fcdhc.2024.1415069

**Published:** 2024-07-10

**Authors:** Emily S. Gitlin, Michelle Demetres, Arthi Vaidyanathan, Nicole Palmer, Hannah Lee, Sabrina Loureiro, Eman Radwan, Abigail Tuschman, Jyoti Mathad, Puja Chebrolu

**Affiliations:** ^1^ Weill Cornell Medicine, New York, NY, United States; ^2^ Duke University School of Medicine, Durham, NC, United States; ^3^ Cornell University, Ithaca, NY, United States; ^4^ Weill Cornell Medicine-Qatar, Ar-Rayyan, Qatar; ^5^ Johns Hopkins University, Baltimore, MD, United States

**Keywords:** gestational diabetes, non-overweight, underweight, normal weight, lean

## Abstract

**Background:**

Gestational diabetes (GDM) affects nearly 15% of pregnancies worldwide and is increasing globally. While this growth is thought to be primarily from overweight and obesity, normal and underweight women are affected as well, particularly in low and middle-income countries. However, GDM in non-overweight women remains understudied. Thus, we examined the prevalence among normal and underweight women globally.

**Methods:**

A comprehensive literature search was performed in Ovid MEDLINE, Ovid EMBASE, and The Cochrane Library. Studies retrieved were screened for eligibility against predefined inclusion/exclusion criteria. Prevalence of GDM among women with normal and underweight body mass index (BMI) was extracted, and average prevalence was calculated globally, by World Health Organization region, and by country. Pregnancy outcomes were described when available.

**Results:**

A total of 145 studies were included. The average global prevalence of GDM among non-overweight women (BMI <25 kg/m^2^) was 7.3% and among underweight women (BMI <18.5 kg/m^2^) was 5.0%. GDM prevalence in non-overweight women was highest in Asia (average 12.1%) and lowest in the African region (0.7%). The countries with the highest prevalence were Vietnam (21.1%), Finland (19.8%), Poland (19.3%), Bangladesh (18.65%), and China (17.7%). The average global prevalence of large for gestational age infants (LGA) born to non-overweight women with GDM was 9.9%, which is lower than the average prevalence in the general population with GDM (14%).

**Conclusion:**

GDM is more common than previously recognized in non-overweight women, particularly in Asia, but also in European countries. Non-overweight women with GDM had lower prevalence of LGA babies compared to prior reported prevalence in all women with GDM, though data on pregnancy outcomes was limited. These findings challenge guidelines that recommend restriction of weight gain for GDM management. Further research on the pathophysiology and complications of GDM in women who are not overweight should be urgently conducted to inform appropriate management guidelines and support optimal pregnancy outcomes.

## Introduction

Gestational diabetes mellitus (GDM) is glucose intolerance that develops during pregnancy, affecting nearly 1 in 7 pregnant women globally (1). The prevalence of GDM is increasing each year in parallel with the type 2 diabetes (T2DM) epidemic. GDM contributes to both short and long-term morbidity and mortality for mother and child. Beyond short-term complications such as preterm birth, pre-eclampsia, and cesarean section, women with GDM also have a 10-fold higher risk of developing T2DM and 2-fold higher risk of future cardiovascular events (2, 3). Similarly, infants born to mothers with GDM not only have a higher risk of large for gestational age (LGA), which can cause birth complications, but also an increased risk of future obesity and glucose intolerance (4–6). Studies of GDM and its complications have largely been done in overweight populations, but recent studies show that GDM is increasing among non-overweight populations as well.

Similar to T2DM, increased weight is a major risk factor for GDM. However, in many low- and middle-income countries, most people with T2DM are underweight or normal weight. Up to 66% of the 200 million South and East Asians with T2DM are underweight or normal weight by BMI (7). It is hypothesized that low birthweight and undernutrition in early life may alter glucose-insulin metabolism in adulthood through genetic variants that have evolved to favor efficient use of nutrients in calorie-limited environments, such as low- and middle-income countries, but also promote T2DM (8–10). A similar pattern and mechanism of non-overweight diabetes in pregnancy may exist but has not been systematically studied. This is important because current World Health Organization (WHO) and International Diabetes Foundation guidelines recommend diet control, exercise, and restriction of weight gain for GDM management, which may not be appropriate for women who have underweight or normal BMI (11, 12).

Understanding the prevalence and pathophysiology of non-overweight women with GDM is important for guiding appropriate screening, management, and follow-up for women and their children. We therefore conducted a scoping review of the scientific literature to investigate the global prevalence and adverse outcomes of GDM among non-overweight women.

## Methods

We performed this study by following the Preferred Reporting Items for Systematic Reviews and Meta-Analyses extension for scoping reviews (PRISMA-ScR). In adherence to this statement, we prospectively registered our protocol with Open Science Framework: https://osf.io/9k7wv/.

### Search strategy

We performed comprehensive searches to identify studies that addressed GDM among normal weight and underweight women.

We ran searches on June 12, 2023, in the following databases: Ovid MEDLINE (ALL - 1946 to Present), Ovid EMBASE (1974 to present), and The Cochrane Library (Wiley). Our search strategy included all appropriate controlled vocabulary and keywords for the concept of “lean gestational diabetes.” The full search strategies for all databases are available in **Additional Supplementary Material A**. To limit publication bias, we did not include language, publication date, or article type restrictions on the search strategy.

### Study selection

**Figure 1:**
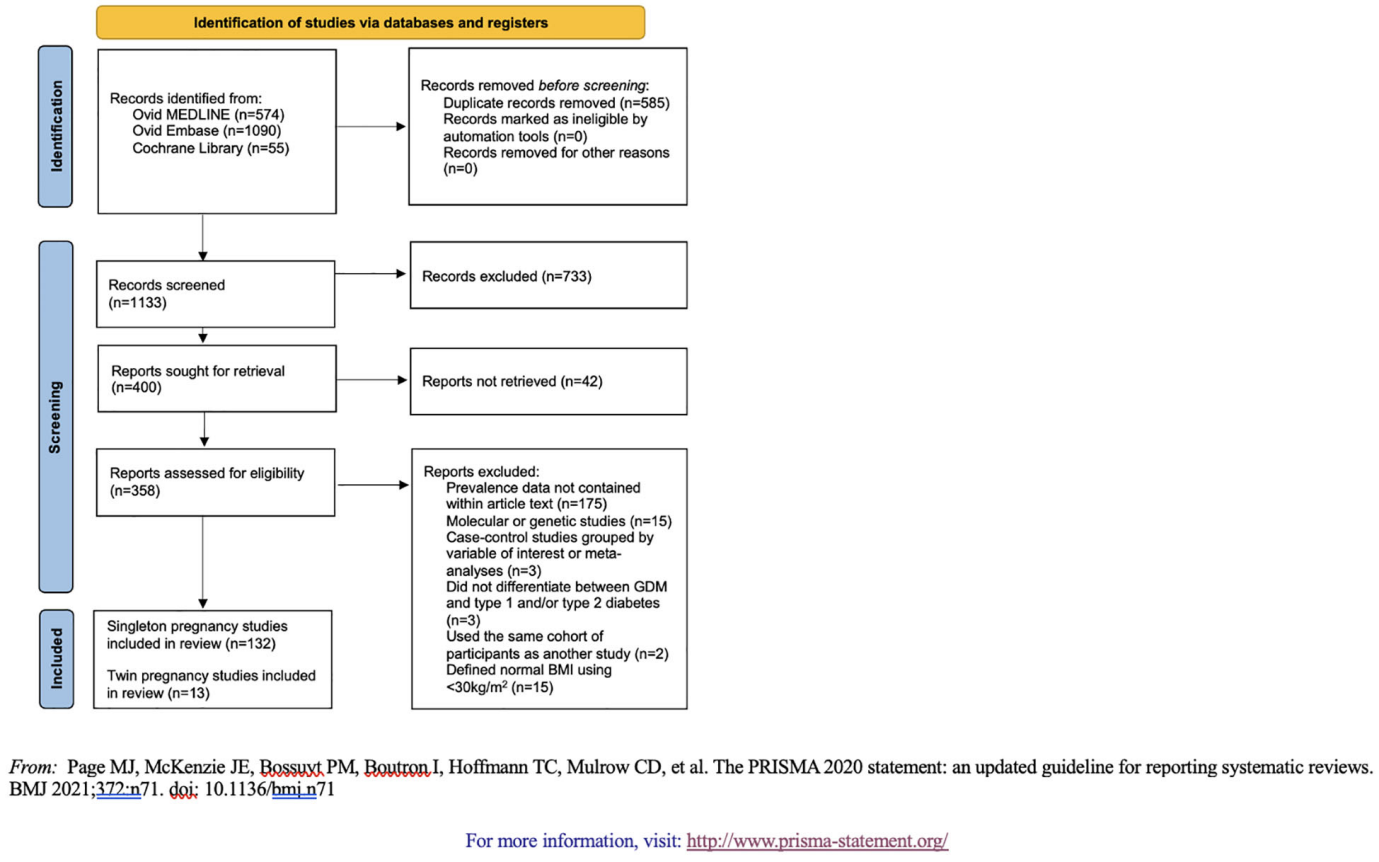
PRISMA 2020 flow diagram of the study.

We screened and retrieved studies for inclusion using Covidence systematic review software. Authors EG, AV, NP, HL, SL, ER, AT, and PC served as independent reviewers. Two independent reviewers analyzed each title and abstract against predefined inclusion/exclusion criteria. Discrepancies were resolved by a third reviewer. For final inclusion, each full text was then retrieved and screened by two independent reviewers. Inclusion criteria for articles were: (1) women ≥15 years of age; (2) data on GDM in women with BMI ≤25 kg/m^2^ (+/- 2kg/m^2^) or mid-upper arm circumference ≤22.7cm; and (3) data on categorical BMI in women diagnosed with GDM (13). Exclusion criteria were: (1) sample size <20; (2) overviews, editorials, case reports, case series, review papers, or method protocols without results; (3) case-control studies where women were selected based on our variables of interest; (4) molecular or genetic studies; (5) studies that did not differentiate between GDM and type 1 and/or type 2 diabetes; (6) studies without access to full-text article; and (7) studies that did not report GDM prevalence data as a fraction or percent within the full text. Case-control studies and studies that did not differentiate between GDM and other types of diabetes were excluded to avoid inaccurate prevalence calculations due to over-sampling. Molecular and genetic studies were also excluded for this reason. Studies that did not report GDM prevalence as a fraction or percent, such as those reported as odds ratios (n=22), were excluded because of an inability to aggregate the data. Excluding these studies might impact the representativeness of our prevalence estimates because we were unable to account for all available data. The full PRISMA flow diagram outlining the study selection process is available in **Figure 1**.

### Data extraction

We performed data extraction independently in duplicate by two independent extractors (authors EG, AV, NP, HL, SL, ER, AT, and PC) with predefined, standardized templates. At the completion of data extraction, each entry was reviewed by a third reviewer, and discrepancies were resolved by source data. Data points defined for extraction were: author(s); publication year; study year(s); total study size; key findings; country of participants; number of study sites; study setting; study design; recruitment and sampling procedures; GDM diagnostic criteria used; GDM medications prescribed; inclusion criteria; prevalence of GDM in women with BMI <25kg/m^2^, <23kg/m^2^, and <18.5kg/m^2^; mean or median age; mean or median BMI and time of measurement; abdominal girth and time of measurement; mid-upper arm circumference and time of measurement; fasting, 1-hour, 2-hour, 3-hour and other glucose levels, time of measurement, and load given; hemoglobin A1C and time of measurement; prevalence of pre-eclampsia among non-overweight women; prevalence of cesarean section among non-overweight women; prevalence of intrauterine growth restriction (IUGR) or small for gestational age (SGA) births among non-overweight women; and prevalence of LGA or macrosomic births among non-overweight women. We also conducted a separate analysis to examine the prevalence of BMI <25kg/m^2^, <23kg/m^2^, and <18.5kg/m^2^ among women with GDM; prevalence of cesarean section among women with GDM; prevalence of IUGR or SGA births among women with GDM; and prevalence of LGA or macrosomia births among women with GDM.

### Data analysis

Extracted data on the prevalence of GDM among normal and underweight women and the prevalence of normal and underweight BMI among women with GDM were analyzed using descriptive statistics, including measures of central tendency and total range. Prevalence of perinatal and postpartum outcomes among women with GDM and trends among the diagnostic criteria used by the extracted studies were also analyzed using measures of central tendency. Prevalence of GDM among women with non-overweight BMIs, prevalence of non-overweight BMI among women with GDM, and prevalence of perinatal and postpartum outcomes were further analyzed by WHO region (14) using measures of central tendency and total range. Analysis of a subgroup of Asian countries from the Western Pacific and South-East Asian WHO regions was also examined. This categorization was done because East and South Asians are known to have a higher risk of diabetes at lower BMI ranges (7).

Studies that included women who gave birth to only twin pregnancies were analyzed as a separate subgroup (**Supplementary Figures 1B**, **2B**; **Supplementary Tables 5**, **9**). However, studies that included both singleton and twin pregnancies were included in the main analysis. Studies that used a case-control method that grouped or selected participants based on one of the outcome variables of interest for this study (GDM status or BMI category) were excluded from prevalence calculations only for the relevant analysis.

## Results

The initial search retrieved 1,133 records which were then screened at the title and abstract level for exclusion criteria; 400 studies were eligible for full-text screening. Of these full texts, 145 were included in the final analyses (**Supplementary Table 1**, **Additional Supplementary Material B**).

An overview of study characteristics and GDM prevalence is shown in **Supplementary Table 1**. The number of participants in each study ranged from 36 to 15 million. Most studies were conducted in the WHO regions of the Western Pacific (n=56), Americas (n=32), and Europe (n=27), with a paucity of data from the African region (n=3). Of the 79 studies that reported which criteria were used to diagnose GDM, International Association of the Diabetes and Pregnancy Study Groups (IADPSG)/WHO 2013 diagnostic criteria were most utilized (n=42, 53.2%), particularly in the Western Pacific and European WHO regions. A smaller number (n=17, 21.5%) used Carpenter-Coustan/American College of Obstetrics and Gynecology diagnostic criteria, primarily in the WHO region of the Americas. Additional details about GDM diagnostic criteria used by the included studies are listed in **Supplementary Table 1**.

When analyzing the collected prevalence data, normal and underweight BMI classifications were not standard across the literature. Thus, within the <25 kg/m^2^ BMI category, we included 18 studies from seven countries in our global prevalence calculations that used BMI cutoffs between 24-27 kg/m^2^ (**Supplementary Tables 2**, **6**). Specifically, some studies from Australia (n=1), China (n=7), Finland (n=1), and United States (n=1) used a BMI cutoff of <24kg/m^2^, while three included studies from the United States and one from Italy selected a BMI cutoff of <26kg/m^2^. Additional studies from Denmark (n=1), Sri Lanka (n=1), and the United States (n=2) used BMI <27 kg/m^2^. Among studies of twin pregnancies, two studies from China used a BMI classification that only included women with BMIs up to <24kg/m^2^ (**Supplementary Tables 5**, **9**). Within the <23 kg/m^2^ BMI category, two studies from China with a <23.9kg/m^2^ BMI cutoff and one study from Spain with a <20.9kg/m^2^ cutoff were included (**Supplementary Tables 3**, **7**). Within the <18.5 kg/m^2^ category, 14 studies from 6 countries with modified cutoffs were included (**Supplementary Tables 4**, **8**). Some studies from India (n=2) and the United States (n=2) utilized a cutoff of <19.8kg/m^2^, while one study from India utilized a cutoff of <19.9kg/m^2^. Additionally, some studies from Finland (n=2), India (n=1), Sweden (n=2), the United Kingdom (n=1), and the United States (n=1) included women with BMIs <20kg/m^2^, and one study from India and one study from Spain included women with BMIs <20.9kg/m^2^.

### Prevalence of GDM among women with non-overweight BMI

**Figure 2:**
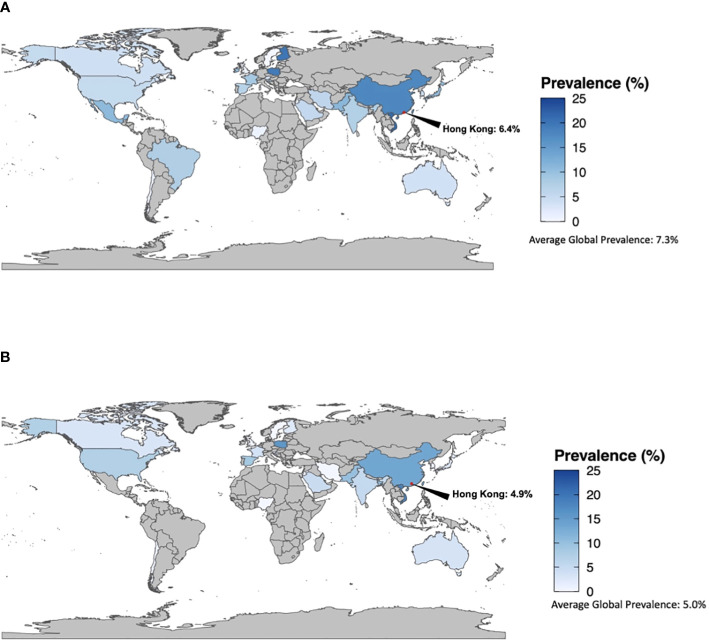
**(A)** Prevalence of GDM among women with BMI <25kg/m^2^. **(B)** Prevalence of GDM among women with BMI <18.5kg/m^2^.

There were 102 total studies from 32 countries and 6 WHO regions that contained GDM prevalence data for pregnant women with BMI <25kg/m^2^. The average global prevalence of GDM among pregnant women with BMI <25kg/m^2^ was 7.3%, ranging from 0.1% in Sweden to 21.1% in Vietnam (**Figure 2A**; **Supplementary Table 2**). By country, the highest prevalences were seen in Japan (average 10.6%, range 0.6-36.4%), Mexico (10.9%), Pakistan (11.2%), China (average 17.7%, range 3.0-34.2%), Poland (19.3%, median BMI in GDM 24.0kg/m^2^, range 21.5–29.1), Finland (average 19.8%, range 5.4-34.2%), and Vietnam (21.1%). Lowest prevalence was seen in Sweden (0.1%), Jordan (0.6%), Nigeria (0.7%), the United Kingdom (average 0.7%, 0.1-1.0%), Chile (1.4%), Turkey (average 2.0%, 0.9-3.1%), and Palau (3.1%). By WHO region, the Western Pacific had the highest prevalence of GDM among pregnant women with BMI <25kg/m^2^ (average 11.8%, range 0.6-36.4%), while the African region had the lowest (0.7%) (**Supplementary Table 1**). Within the subgroup of Asian countries, average GDM prevalence in women with BMI <25 kg/m^2^ was 12.1% (range 0.0-36.4%).

There were 11 studies from six countries, primarily in Asia, that reported data for pregnant women with BMI <23kg/m^2^ (Bangladesh (n=2), China (n=3), India (n=3), Singapore (n=1), South Korea (n=1), Croatia (n=1)). The average global prevalence of GDM in this group was 12.5%, ranging from 3.5% in South Korea to 18.7% (10.6-26.7%) in Bangladesh (**Supplementary Figure 1A**, **Supplementary Table 3**).

There were 85 studies from 29 countries and 6 WHO regions that reported data on pregnant women with BMI <18.5 kg/m^2^. The average global prevalence of GDM for women in this group was 5.0% (**Figure 2B**; **Supplementary Table 4**), ranging from 0% in Nigeria, Iran, and Sweden to 18.3% in Vietnam. The United States (average 7.3%, 0.0-73.6%), Spain (average 8.7%, 2.3-21.4%), Pakistan (10.0%), Bangladesh (10.4%), China (average 11.7%, 1.4-18.2%), Poland (15.8%), and Vietnam (18.3%) had the highest prevalence of GDM. Nigeria (0.0%), Iran (0.0%), Sweden (0.0%), Jordan (0.1%), United Kingdom (average 0.3%, 0.0-0.6%), Chile (0.9%), and Turkey (average 1.3%, 0.0-2.7%) had the lowest. By WHO region, the Western Pacific had the highest prevalence of GDM among pregnant women with BMI <18.5kg/m^2^ (average 8.7%, 0.0-18.3%), while the African region had the lowest (0.0%) (**Supplementary Table 1**).

For analysis of twin studies (n=12), data are included in **Supplementary Figure 1B**, **Supplementary Table 5**. The average prevalence of GDM in twin pregnancies was 11.7% (5.1-20.8%) among women with normal or underweight BMI (<25kg/m^2^).

### Prevalence of non-overweight BMI among women with GDM

**Figure 3:**
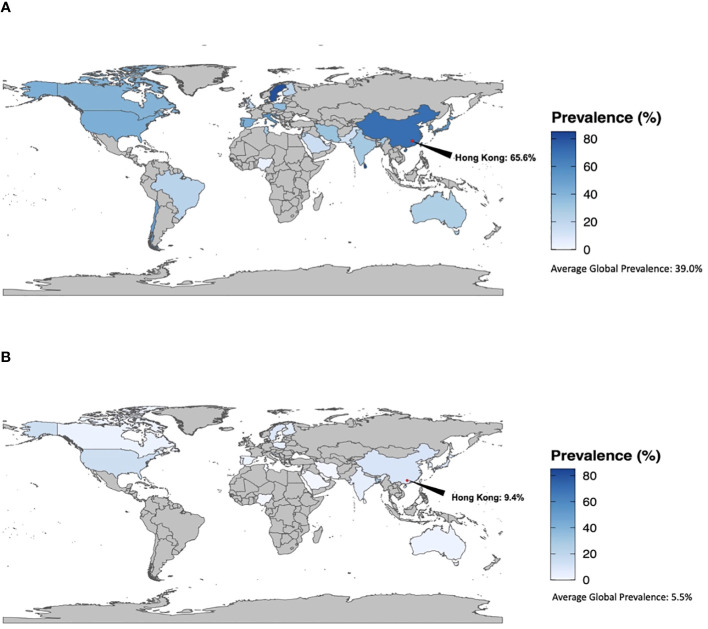
**(A)** Prevalence of BMI <25kg/m^2^ among women with GDM. **(B)** Prevalence of BMI <18.5kg/m^2^ among women with GDM.

Average prevalence of normal and underweight BMI among women diagnosed with GDM is shown in **Figures 3A, B**; **Supplementary Figure 2A**. The average prevalence of BMI <25kg/m^2^ and <23kg/m^2^ among women with GDM was 39.0% (3.3-80.5%) and 39.5% (15.2-61.7%), respectively, while 5.5% (0.0-27.6%) of women with GDM had a BMI of <18.5kg/m^2^. BMI <25kg/m^2^ was most prevalent among women with GDM in the Western Pacific WHO region (average 62.8%, 3.7-89.7%) and least prevalent in the African region (average 10.9%, 0.8-28.8%) (**Supplementary Table 1**). Conversely, BMI <18.5kg/m^2^ among women with GDM was most prevalent in the WHO region of the Americas (average 10.8%, 0.0-56.7%) and least prevalent in the Eastern Mediterranean region (average 0.2%, 0.0-0.4%). The South-East Asian and Western Pacific WHO regions had moderate prevalence at 8.2% (0.0-27.6%) and 8.1% (0.4-17.4%), respectively. In twin studies (n=9), the average prevalence of BMI <25kg/m^2^ among women with GDM was 73.7% (60.9-85.9%) (**Supplementary Figure 2B**, **Supplementary Table 9**).

### Outcomes associated with GDM by BMI

Twenty-four studies included data on perinatal and postpartum outcomes associated with GDM (**Table 1**). Among non-overweight women with GDM, the average prevalence of pre-eclampsia was 4.9% (0.2-15.0%). In comparison, a scoping review of singleton women of any BMI with GDM found prevalence to be 9.6% (15). By WHO region, the rate of pre-eclampsia ranged from 1.7% in the Western Pacific to 8.1% in the European region. Additionally, an average of 35.2% (3.2-72.7%) of women with non-overweight BMI and GDM underwent cesarean section (36.4% among all BMIs) (15). The prevalence of cesarean sections among non-overweight women with GDM was lowest in the Western Pacific WHO region (34.7%, 3.2-54.7%) and highest in the South-East Asian WHO region (72.7%). Furthermore, the overall prevalence of SGA in non-overweight women with GDM was 7.0% (0-19.2%) (it was 7.3% in a comparison review of all BMIs) (15). Regionally, SGA in women with GDM ranged from 5.1% in the Western Pacific WHO region to 9.5% in the European WHO region. Lastly, the overall prevalence of LGA in infants born to non-overweight women with GDM was 9.9% (0.4-33%) (in comparison to 16.3% among women of all BMIs) (15). The Western Pacific WHO region had the lowest prevalence at 6.2%, while the WHO region of the Americas had the highest prevalence at 14.3%. There were no studies from the African WHO region that included perinatal and postpartum outcomes for non-overweight women with GDM.

**Table 1:** Average prevalence of adverse perinatal and postpartum outcomes among normal and underweight women with GDM.

Adverse Outcome	Average Prevalence						
	African Region (AFR)	Region of the Americas (AMR)	South-East Asian Region (SEAR)	European Region (EUR)	Eastern Mediterranean Region (EMRO)	Western Pacific Region (WPRO)	Overall
Pre-eclampsia	NA	1.4%	NA	8.1%	NA	1.7%	4.9%
C-section	NA	25.0%	72.7%	35.4%	NA	34.7%	35.2%
IUGR/SGA	NA	NA	NA	9.5%	NA	5.1%	7.0%
LGA/Macrosomia	NA	14.3%	NA	10.0%	11.1%	6.2%	9.9%

NA: Not Applicable.

## Discussion

In this scoping review, we found that the average global prevalence of GDM in non-overweight women was 7.3%. The highest average prevalence among non-overweight women was seen in Asia (12.1%, 0.0-36.4). However, some countries in Europe had unexpectedly high prevalences of up to 19.3%. We also found that the prevalence of LGA infants among non-overweight women with GDM from the Western Pacific WHO region was lower compared to the prevalence of LGA in Europe and the Americas (average 6.2% vs 10.0% and 14.3%, respectively). Our findings suggest that GDM is a significant issue among women without overweight BMIs, especially in Asia, and current GDM management guidelines may not be targeted to protect the health of mothers and their children in all populations.

In the non-pregnant adult population, South and East Asians are known to have a high risk of diabetes at non-overweight BMIs (7). Diabetes is caused by both excessive insulin resistance and by the body’s inability to produce enough insulin to compensate for the insulin resistance (16). Insulin resistance in South and East Asians often stems from the inflammation caused by central adiposity and elevated body fat, which do not always correlate with total body weight. Insulin deficiency, however, may be even more important than insulin resistance in the Asian population (17). The Barker hypothesis posits that *in utero* undernutrition predisposes the fetus to impaired pancreatic growth and insulin deficiency after birth (8). The fetus/child has an increased risk of diabetes itself, and female children may be predisposed to GDM when they become pregnant later in life because of the metabolic stress of pregnancy on their underdeveloped pancreas. This cycle contributes to the exponential increase of GDM and type 2 diabetes in Asia. Undernutrition, particularly of protein and micronutrients, is present in the majority of South Asians (18, 19). Heterogeneity in GDM prevalence across geographies may also be explained by cultural, dietary, genetic, and economic factors as well. In African regions, genetic factors and lower healthcare access may be contributing to lower prevalence of GDM in the underweight even though there is a high prevalence of undernutrition (20, 21). Dietary and cultural factors also influence risk. A study of Asian people in the United States with GDM, for example, found that greater acculturation was protective against GDM (22). Cultural changes in diet and exercise may contribute to this change in risk.

Overall, studies from the European region reported a lower average prevalence of GDM in women with BMI <25kg/m^2^ (7.3%, 0.1-34.2%) than those from Asia. However, in some European countries, the prevalence was very high. In Poland and Finland, 19.3% and 19.8% of women who were normal or underweight, respectively, had GDM. Studies in both countries predominately or only recruited Caucasian women. The high prevalence appears to be driven by cases in the normal weight rather than the underweight group. In Poland, the median BMI among women diagnosed with GDM was 24.0kg/m^2^. The higher prevalence in Poland and Finland may reflect their use of the IADPSG criteria for GDM screening. IADPSG has been shown to have increased sensitivity compared to other criteria (e.g. Carpenter-Coustan, O’Sullivan) because of lower diagnostic thresholds. Furthermore, studies from Denmark suggest that IADPSG criteria may be more sensitive for detecting GDM among normal weight women, who have increased body fat without increased BMI (23, 24). IADPSG criteria are recommended because of their association with adverse GDM outcomes (6). While Poland and Finland noted higher GDM prevalence with IADPSG, countries with national guidelines recommending risk factor-based screening (i.e. Sweden, France, the United Kingdom) had lower prevalences of GDM. Our results suggest that with appropriate screening, GDM among non-overweight women is not restricted to people of Asian ancestry.

Few studies reported perinatal or postpartum outcomes associated with GDM in women of non-overweight BMI, but there were still important differences in outcomes compared to prior GDM research. In the current literature, LGA is the most common adverse pregnancy outcome of GDM and is thought to lead to other complications such as cesarean section, neonatal hypoglycemia, stillbirth, and birth asphyxia (6). In overweight people, insulin resistance in GDM increases glucose, cholesterol, and protein availability to the fetus and leads to fetal hyperinsulinemia. Fetal hyperinsulinemia, in turn, promotes fetal growth. Consequently, over 16% of women with GDM have LGA babies (15). However, in our study, we found that the prevalence of LGA was only 6.2% in the Western Pacific WHO region, which is dominated by studies from China. Asian populations may have a stronger component of insulin deficiency rather than insulin resistance which may contribute to limited fetal growth. More data on outcomes such as pre-eclampsia, SGA, and LGA in non-overweight women with GDM should be collected in Southeast Asian and African countries to guide appropriate management.

We also found that the prevalence of SGA among non-overweight women with GDM was 7.0%. Studies from Jordan, Palau, and Portugal reported much higher prevalence of SGA in infants of non-overweight women (10.6%, 11.7%, and 19.2%, respectively). Women with GDM can be at risk for fetal growth restriction. Infants of non-overweight women may not be exposed to lipid excess or hyperinsulinemia, and therefore may be predisposed to growth restriction instead of overgrowth. Thus, treatment guidelines which focus on reducing factors that cause insulin resistance (such as limiting total carbohydrate intake and restricting gestational weight gain) may potentially contribute to the increased prevalence of SGA babies in mothers with low BMI and GDM. Similarly, smaller placental size and lower vascularity may be present in non-overweight women, further limiting nutrient delivery to the fetus (25, 26). Unlike pre-eclampsia, which also limits placental size and vascularity, there are no guidelines for optimal timing of delivery in GDM. Evidence based guidelines for normal and underweight women with GDM are urgently needed to prevent potential harm from current GDM management guidelines, which are primarily focused on overweight women.

Our study had several strengths and limitations. First, to our knowledge, we conducted the first scoping review of GDM prevalence in non-overweight women. Second, we followed a thorough and broadly inclusive screening and extraction process which was geographically diverse. We did not exclude studies based on the text’s language, and we included all studies with available data, not just studies that aimed to describe prevalence among non-overweight women. However, we did not separate prevalence by method of GDM screening, potentially allowing for over- or underdiagnosis of GDM in certain populations due to heterogeneity in diagnostic criteria. Since BMI ranges varied, we also included some studies which had a BMI range above or below our prescribed normal weight and underweight cutoffs. This allowed inclusion of more studies in our analysis but limits the absolute comparability of prevalence data. Furthermore, we were unable to assess racial and ethnic background of participants in all studies, possibly limiting the generalizability of our prevalence data for each country. Conversely, we did obtain data on multiple other variables, including pregnancy outcomes, that have been infrequently reported and that may guide future studies on GDM outcomes in non-overweight women.

## Conclusions

GDM is more common than previously recognized in non-overweight women, particularly in Asia, but also in European countries. Further research on the pathophysiology and complications of GDM in women who are not overweight should be urgently conducted to inform appropriate management guidelines and support optimal pregnancy outcomes.

## Data availability statement

The original contributions presented in the study are included in the article/**Supplementary Material**. Further inquiries can be directed to the corresponding author.

## Author contributions

EG: Data curation, Formal analysis, Project administration, Supervision, Visualization, Writing – original draft, Writing – review & editing, Investigation. MD: Methodology, Project administration, Software, Writing – review & editing. AV: Data curation, Formal analysis, Investigation, Writing – original draft, Writing – review & editing. NP: Data curation, Formal analysis, Investigation, Writing – original draft, Writing – review & editing. HL: Data curation, Formal analysis, Investigation, Visualization, Writing – original draft, Writing – review & editing. SL: Data curation, Formal analysis, Investigation, Visualization, Writing – original draft, Writing – review & editing. ER: Data curation, Formal analysis, Investigation, Visualization, Writing – original draft, Writing – review & editing. AT: Data curation, Formal analysis, Investigation, Visualization, Writing – original draft, Writing – review & editing. JM: Conceptualization, Funding acquisition, Supervision, Writing – review & editing. PC: Conceptualization, Data curation, Formal analysis, Funding acquisition, Investigation, Methodology, Project administration, Supervision, Writing – original draft, Writing – review & editing.
