# Supplementary material for: The prevalence of gestational diabetes among underweight and normal weight women worldwide: a scoping review
Source: Front Clin Diabetes Healthc. 2024 Jul 10;5:1415069. doi: 10.3389/fcdhc.2024.1415069 (PMC11266185; doi:10.3389/fcdhc.2024.1415069)
Supplement: Supplementary file 1 [file DataSheet_1.pdf]

Supplementary Table 1. Characteristics of included studies. *Study included women with twin pregnancies. **BMI taken pre-pregnancy.

Author	Total population (n)	Country [City]	GDM Diagnostic Criteria	Study Population	Prevalence of GDM among non-overweight women		Prevalence of non-overweight among women with GDM	
					<25 kg/m^2	<18.5 kg/m^2	<25 kg/m^2	<18.5 kg/m^2
**African Region (AFRO)**					0.7%	0.0%	10.9% (0.8-28.8%)	0.7% (0.0-1.3%)
Abadia et al., 2021	528	Tunisia	WHO'99	Pregnant women with GDM			28.8%	1.3%
Ahmed et al., 2009	122	Egypt [Sohag]	WHO '99	All pregnant women at Sohag University Hospital			0.8%	
Senbanjo et al., 2021	365	Nigeria [Ifako-Iljaye, Lagos]	US IoM categorization	Women with singleton pregnancy	0.7%	0.0%	3%	0%
**Region of the Americas (AMRO)**					4.6% (0.2-21.1%)	2.4% (0.0-7.4%)	39.7% (14.0-92.5%)	10.8% (0.0-56.7%)
Al-Obaidly et al., 2014*	504	Canada [Toronto]	Carpenter-Coustan/ACOG	Women delivering viable twins	6.0%	9.1%		
Relph et al., 2021	703,115	Canada [Ontario]		All pregnancies in the Ontario birth registry	3.9%	2.8%	39.0%	2.8%
Lipworth et al., 2021*	1,274	Canada [Toronto, Ontario]		Twin pregnancies in in a tertiary care center in Toronto	4.1%	0.0%		
Vigneault et al., 2015	299	Canada [Quebec City]	Canadian Diabetes Association	Pregnant women in the Quebec provincial registry with and without GDM			40.0%	
Akinyemi et al., 2022	15,627,572	USA		Pregnant women in the US Vital Statistics registry	3.7%	2.9%		
Li et al., 2020	321	USA	Carpenter-Coustan/ACOG	Non-obese controls with singleton pregnancies			34.6%	
Houser et al., 2011	61,726	USA	Carpenter-Coustan/ACOG	Women in a perinatal database	0.2%	0.0%	14.0%	0.0%
Amini et al., 2010	9,227	USA [Maryland, DC, Virginia]		All women delivering in Medstar Health hospitals.	1.9%	0.8%	23.4%	0.8%

Khalak et al., 2015	2,155	USA [New York]		All women in the New York State Perinatal Data System	3.7%	2.0%	22.3%	1.1%
Hollingsworth et al., 1991	7,895	USA [San Diego, California]	O'Sullivan	Women delivering at a tertiary care center			(45.8%)	
He et al., 2012	96	USA [Rhode Island]	Carpenter-Coustan/ACOG	Non-overweight and overweight pregnant women	4.4%			
Lowry et al., 2018	2,664,126	USA [Florida]		All women in the Florida Birth Defects Registry with a live birth	2.6%	2.1%	32.6%	2.1%
Rudra et al., 2007	1,644	USA [Washington]		Primarily nulliparous women enrolled from clinic	3.9%		92.5%	56.7%
Baeten et al., 2001	96,801	USA [Washington]		Singleton deliveries in the Washington birth certificate tapes	1.5%		11.3%	49.1%
Feresu et al., 2015	255,773	USA [Indiana]		Mothers in the Indiana State Department of Health (ISDH) birth registry	2.5%	2.1%	22.6%	1.1%
Mackeen et al., 2019	27,523	USA [Pennsylvania		Singleton pregnant women delivering at a hospital	3.4%	2.4%		
Schuster et al., 2015	6,907	USA [Northeast Pennsylvania]		Women with singleton births delivering at a hospital	13.5%		32.8%	
Ogunyemi et al., 1998	582	USA [Alabama]	Carpenter-Coustan/ACOG	Low-income, Black pregnant women with singleton deliveries in western Alabama	1.3%		26.7%	0.0%
Shin & Song, 2015	219,868	USA [40 States]		All women in the Pregnancy Risk Assessment Monitoring System with live-born infants	6.5%	6.0%	39.5%	3.5%
Thayer et al., 2021	893,965	USA [Portland]		Multiparous women without prior Cesarean delivery, having singleton, non-anomalous, term pregnancies	9.7%	7.4%	45.3%	2.3%

				within the period of 2007 to 2011.				
VivianUkah et al., 2019	722,839	USA [Washington State]		Women with singleton pregnancies in Washington state	3.6%	0.2%	29.3%	1.5%
Yao et al., 2017	2.3 million	USA [Texas]		Women with singleton birth in Texas	3.0%			
Catalano et al., 1993	390	USA [Vermont]	Carpenter-Coustan/ACOG	Women with singleton pregnancies having GDM			62.8%	19.2%
Chen et al., 2010	2,379	USA [New Jersey]	Carpenter-Coustan/ACOG	Women with singleton pregnancies			79.6%	
Cheney et al., 1985	41	USA [San Diego, California]	O'Sullivan	Nonobese women (pre-pregnancy BMI <24)			65.7%	
Song et al., 2022	152	USA [Amarillo, Texas]		Han Chinese women with singleton pregnancies delivered via cesarean section			47.1%	
Ehrenberg et al., 2004**	12,303	USA [Cleveland, Ohio]	Carpenter-Coustan/ACOG	Singleton pregnancies in the perinatal database of an urban tertiary health center	1.6%	1.5%		
Garmendia et al., 2018	62,579	Chile [Santiago]	Chilean Ministry of Health guidelines	Singleton deliveries at a hospital	1.4%	0.9%		
Olmos et al., 2012	251	Chile [Santiago]		Women with GDM referred for specialist care			49.8%	
Rocha et al., 2020	154	Brazil [Porto Alegre City]	IADPSG/WHO '13	Pregnant women at Murialdo Teaching Health Center	7.6%		22.2%	
Romero Gutierrez et al., 2006	684	Mexico		Pregnant women with pre-pregnancy BMI 18.5-24.9	21.1%			
Zonana-Nacach et al., 2010	1,000	Mexico [Tijuana]		Women delivering at a tertiary hospital	0.7%			

**Eastern Mediterranean Region (EMRO)**					6.8% (0.6-17.1%)	5.0% (0.0-10.0%)	23.2% (8.3-45.0%)	0.2% (0.0-0.4%)
Chodick et al., 2021	190,905	Israel	Carpenter-Coustan/ACOG	Pregnant women registered in the Israeli Defense Forces and Maccabi Healthcare Services (MHS) databases	5.1%	6.1%		
Meher-Un-Nisa et al., 2009	1,000	Saudi Arabia [Buraida]		All pregnant women admitted for routine or emergency care	3.2%		18.9%	
Wahabi et al., 2021	7,029	Saudi Arabia [Riyadh]	WHO	Singleton pregnant women in 3 hospitals	12.0%	8.9%		
Kumari, 2001	188	United Arab Emirates [Abu Dhabi]		Singleton normal weight pregnant women as a control group to obese women			8.3%	
Hantoushzadeh et al., 2016	1,279	Iran [Tehran]	Carpenter-Coustan/ACOG	Singleton pregnant women at a tertiary care center	5.8%	0.0%	45.0%	0.0%
Hashemipour et al., 2018	319	Iran [Qazvin]	IADPSG/WHO '13	Singleton pregnant women with GDM			19.7%	
Karasneh et al., 2021	22,591	Jordan		All pregnant women in national perinatal mortality survey	0.6%	0.1%	29.6%	0.4%
Asad et al., 2022	164	Pakistan [Punjab]		All pregnant women	11.4%		13.2%	
Farooq et al., 2022	230	Pakistan [Sindh]		Singleton pregnancies	5.2%	10.0%		
Jahan et al., 2022	82	Pakistan [Karachi]		Singleton matched non-obese controls	17.1%			
AbuYaacob et al., 2002**	150	Qatar [Doha]		Singleton matched non-obese controls			25.7%	
**European Region (EURO)**					7.3% (0.1-34.2%)	5.6% (0.0-21.4%)	37.9% (4.3-80.6%)	5.0% (0.6-15.2%)
AlvarezCuenod et al., 2010	1,407	Spain [Gran Canaria]		Women with a singleton pregnancy	4.1%	2.5%	31.9%	2.2%
Martinez-Frias et al., 2005	12,489	Spain	O'Sullivan	All pregnant women	3.3%		59.9%	15.2%

Perea et al., 2022	1,036	Spain [Barcelona]	NDDG	Singleton with GDM	12.3%	21.4%	38.1%	1.7%
Pathi et al., 2006	200	United Kingdom		Singleton matched nonobese controls	1.0%		7.1%	
Anand et al., 2011	33,509	UK [Manchester]		All inpatient deliveries	1.0%			
Sebire et al., 2001	215,105	UK [North West Thames Region]		All delivered women in the St Marys' Maternity Information System database	0.7%			
Min et al., 2004	114	UK [London]	EASD Criteria 1979	Singleton pregnant women at an antenatal clinic			28.3%	
CarducciArtenisio et al., 1999	261	Italy	Carpenter-Coustan/ACOG	Pregnant women who screened positive for GDM			55.8%	
Delmis et al., 2015	4,646	Croatia [Zagreb]	IADPSG/WHO '13	All pregnant women	14.7% (12.7%)	7.4%	56.0%	3.4%
Vince et al., 2021	32,051	Croatia	Croatian Society for Gynaecology and Obstetrics	Singleton pregnancies	3.3%	2.8%	54.0%	3.3%
Denison et al., 2014	12,280	Scotland		All singleton deliveries in Scotland	0.1%	0.0%	16.9%	
Ducarme et al., 2007	5,686	France	IADPSG/WHO '13	All women who delivered at a single hospital	10.9%			
Salmon et al., 2021	14,246	France [Normandy]		Singleton pregnant women	6.2%	3.5%		
ChenXu & Coelho, 2022	13,467	Portugal	IADPSG/WHO '13	Pregnant women diagnosed with gestational diabetes or diabetes mellitus			43.1%	1.9%
Machado et al., 2020	3,103	Portugal	IADPSG/WHO '13	Singleton pregnancies of women diagnosed with gestational diabetes			43.2%	
Simoes et al., 2017*	1,794	Portugal [Lisbon]	Carpenter-Coustan/ACOG	Twin pregnancies diagnosed with gestational diabetes mellitus			78.3%	
McGoldrick et al., 2013	499	Ireland		All pregnant women	10.4%		14.0%	
Lewandowska, 2021	912	Poland	IADPSG/WHO '13	All singleton pregnant	19.3%	15.8%	6.8%	0.6%

				Caucasian women				
Ogonowski et al., 2009	2,132	Poland [Szczecin]	WHO	Women diagnosed with gestational diabetes mellitus			68.2%	10.9%
Lindholm et al., 2015	71,638	Sweden		Primiparous singleton pregnant women in the Swedish Medical Birth Register	0.1%		80.6%	8.3%
Lucovnik et al., 2014	4,088	Slovenia	2009 IOM guidelines	Women with GDM			48.2%	2.1%
Lucovnik et al., 2018	271,913	Slovenia	IADPSG/WHO '13 & Carpenter-Coustan/ACOG	Singleton pregnancies	5.8%	2.1%		
TrojnerBregar et al., 2017	157,150	Slovenia	Slovenian Guidelines	Women in the Slovenian National Perinatal Information System	2.0%	1.3%	4.3%	4.3%
Ravnsborg et al., 2016	407	Denmark [Odense]	IADPSG/WHO '13	Singleton pregnancy and HbA1c values of <6.5% at the time of GDM diagnosis.			32.2%	
Kong et al., 2019	649,043	Finland		All pregnancies ending in live births in the Finland Drugs and Pregnancy database.	34.2%	1.2%	8.3%	5.0%
Holopainen et al., 2023	328,892	Finland	Finnish Medical Society	All pregnant women with singleton pregnancies in Finland	5.4%	3.8%	32.7%	5.9%
Kleinwechter et al., 2022	1,490	Austria [Linz]; Germany	IADPSG/WHO '13	Pregnant women with clinically confirmed COVID-19	4.4%		25.7%	
**South-East Asia Region (SEARO)**					6.8% (0.0-24.4%)	7.6% (0.0-13.6%)	33.4% (0.6-78.0%)	8.2% (0.0-27.6%)
Anchala & Ruchi, 2021	1,000	India [Uttar Pradesh]		Women with singleton pregnancy delivering at a hospital	24.4%	11.8%	60.0%	7.1%
Chaurasia & Dwedi, 2021	100	India [Gaya, Bihar]		Women with singleton	3.3%	10.0%	40.0%	20.0%

				pregnancy at a clinic				
De et al., 2023	1,396	India [New Delhi]	IADPSG/WHO '13	All women with singleton pregnancy delivering in a hospital	14.7% (12.7%)	7.4%	59.7% (35.6%)	
Kutchi et al., 2020	200	India [Tamilnadu]		Pregnant women enrolled in clinic	10.0%		22.2%	
Mandal et al., 2011	844	India [Kolkata]		Pregnant women with singleton pregnancies.			(16.3%)	
Naik et al., 2022	3,940	India [Bambolim]		Singleton pregnancies delivering in a hospital	0.2%	0.0%	0.6%	0.0%
Sahu et al., 2007	380	North India		Singleton pregnancies delivering at a tertiary care center	0.8%		22.2%	0.0%
Somani et al., 2022	127	India [Japiur, Rajasthan]		Primigravid women with singleton pregnancy from a clinic	0.0%	0.0%		
Tripathi et al., 2021	100	India		Women with singleton pregnancy in an antenatal clinic	6.0%			
Trivikrama et al., 2023	199	India [Kozhikode, Malappuram, and Wayanad]		Women with singleton pregnancy enrolled at delivery	20.1%	13.6%		
Verma & Shrimali, 2012	784	India [Udaipur and Rajasthan]		Women with singleton pregnancies	0.2%		8.3%	0.0%
Madhavan et al., 2008	106	India [Kottayam, Kerala]	Carpenter-Coustan	Women with singleton pregnancy	(2.8%)			
Madhavi et al., 2022	200	India [Kadapa]		Matches non-obese control women	2.0%			
Bhowmik et al., 2019	498	Bangladesh [Dhaka]	IADPSG/WHO '13	Primigravid singleton pregnancies without a prior history of diabetes	(10.6%)	10.4%	(72.4%)	27.6%
Fakhrul-Alam et al., 2020	80	Bangladesh	IADPSG/WHO '13	Singleton pregnancy enrolled in clinic	(26.7%)		10.0%	

Vidanalage et al., 2016	435	Sri Lanka		Pregnant mothers admitted to a hospital			78% (27%)	3.0%
**Western Pacific Region (WPRO)**					11.8% (0.6-36.4%)	8.7% (0.0-50.9%)	62.8% (3.7-89.7%)	8.1% (0.4-17.4%)
Akgol et al., 2020	496	Turkey [Diyarbakir]		Pregnant women admitted for delivery.				
Avci et al., 2015	931	Turkey [Istanbul]		Pregnant women attending antenatal clinic	0.9%	0.0%		
Ekin et al., 2017	809	Turkey [Izmir]		Singleton pregnancies monitored in the postnatal clinic.	3.1%	2.7%	51.3%	5.1%
Cai et al., 2021	123	Singapore	WHO '99	Pregnant women enrolled in clinic	11.4%			
He et al., 2022	704	Singapore	IADPSG/WHO '13	Singleton pregnant women enrolled from clinic	5.4%	5.1%	43.9%	5.3%
Loy et al., 2016	985	Singapore		Women of Chinese with Malay or Indian ethnicity	(14.4%)			
Feng & Huang, 2021	3,531	China [Guangzhou]		Singleton pregnancies enrolled in clinic	20.8%	14.3%	80.2%	13.3%
Gao et al., 2023*	2,857	China [Chongqing]	IADPSG '10	Twin pregnancy delivering at a hospital	23.8%	16.0%	72.5%	7.2%
Gu et al., 2023	5,614	China [Guangzhou]	IADPSG/WHO '13	Singleton pregnant women enrolled from clinic	17.6%	13.2%	85.2%	13.1%
Hu et al., 2021	945	China [Shenyang]	IADPSG/WHO '13	Mothers and infants who had participated in the 'Born in Shenyang Cohort Study.'	18.8%	9.0%	64.4%	5.9%
Jia et al., 2018	5,305	China [Beijing]		Pregnant women admitted from clinic	(18.5%)	17.6%		
Lan et al., 2020	1,910	China [Southwest China]	IADPSG/WHO '13	Pregnant women admitted from clinic	34.2%	17.9%	84.0%	9.7%
Li et al., 2015	2,488	China [Beijing]	ADA	Singleton pregnant women	11.8%	8.4%	67.5%	10.8%
Lin et al., 2019*	586	China [Foshan]		Twin pregnancies	18.4%	9.9%		

Liu et al., 2015	2,973	China [Beijing]	IADPSG/WHO '13	Primigravid singleton pregnancies	22.8%	15.0%	71.0%	5.5%
Liu et al., 2011	5,047	China [Shenyang]		Primigravid singleton pregnancies	3.0%	1.4%	49.3%	3.5%
Lyu et al., 2023	11,168	China	IADPSG/WHO '13	Singleton pregnant women with GDM			68.5%	7.3%
Schaefer et al., 2018	8,381	China [Guangzhou]	IADPSG/WHO '13	Singleton pregnant women enrolled from clinic	12.8%	9.1%	89.7%	17.4%
Sun et al., 2014	1,418	China [Shanghai]	IADPSG/WHO '13	Pregnant women with GDM			61.8%	7.6%
Tang et al., 2021	1,757	China [Chengdu]	IADPSG/WHO '13	Singleton pregnancies enrolled after delivery			28.2%	
Teshome et al., 2021	3,253	China [Wuhan]	IADPSG/WHO '13	All pregnant women	7.9%	6.4%	78.2%	13.7%
Wang et al., 2020*	312	China		Twin pregnancies with two live births	27.0%	17.0%	85.9%	14.1%
Wang et al., 2018	622	China		Singleton pregnancies with GDM			76.2%	10.6%
Wang et al., 2021	6,223	China	IADPSG/WHO '13	Women with singleton pregnancy without known diabetes	(19.9%)	17.0%	(71.6%)	16.4%
Wang et al., 2018	21,577	China [Beijing, Gaunzghou, Chengdu]	China's Ministry of Health criteria	Women with singleton pregnancy	17.4%			
Wei et al., 2015*	14,451	China [Beijing]	IADPSG/WHO '13	Pregnant women enrolled after delivery	17.7%	13.0%	77.4%	5.8%
Wei et al., 2019	34,087	China [Beijing, Gaunzghou, Chengdu]	IADPSG/WHO '13	Singleton pregnant women	11.7%		(71.4%)	
Wen et al., 2020*	324	China [Chongqnig]	IADPSG/WHO '13	Pregnant women undergoing 75 g oral glucose tolerance test	(28.7%)	7.7%	73.3%	3.0%
Wu et al., 2022	1,289	China [Shanghai]	IADPSG/WHO '13	All women receiving antenatal care		6.1%	70.1%	5.9%
Xintong et al., 2022	6,698	China [Zhengzhou]	IADPSG '10	Singleton pregnant women without known diabetes	11.0%		41.1%	

Xiong et al., 2022	6,598	China [Xiamen City]	IADPSG/WHO '13	Pregnancies achieved by reproductive assistant treatment	24.6%	18.2%	82.9%	9.2%
Yang et al., 2023	6,174	China	IADPSG/WHO '13	All pregnant women	(13.0%)	9.3%	68.3%	8.4%
Zhang et al., 2020	22,223	China [Chongqnig]	IADPSG/WHO '13	Women with singleton pregnancies and no preexisting diabetes	23.0% (20.4%)	13.8%	71.6% (51.8%)	7.2%
Zhao et al., 2021	1,108	China [Zhengzhou]	WHO	Women with a singleton pregnancy	24.6%	2.3%	76.3%	7.1%
Zhao et al., 2021*	369	China [Wenzhou]	IADPSG/WHO '13	Pregnant women without known diabetes.		15.8%	75.3%	13.6%
Zheng et al., 2022	14,578	China	IADPSG/WHO '13	Women with GDM			86.8%	13.9%
Zhou et al., 2020	8,169	China [Foshan]	IADPSG/WHO '13	Singleton pregnancies with no diabetes prior to pregnancy	13.6%	9.8%	70.2%	9.7%
Chung, 2022	465	Hong Kong		Chinese women with singleton pregnancy	6.4%	4.9%	65.6%	9.4%
Cunningham & Teale, 2013	6,138	Australia [Victoria]	IADPSG	All mothers in the Australasia Diabetes in Pregnancy system.	2.8%	3.0%	18.9%	0.9%
Foo et al., 2016	55,352	Australia [Brisbane]		Singleton deliveries	5.7%	5.5%	3.7%	0.4%
Knight-Agarwal et al., 2016	14,875	Australia		Mothers with singleton pregnancies at the hospital.	1.6%	1.2%		
McIntyre et al., 2012	75,432	Australia [Brisbane]		Women with singleton pregnancies	1.2%	1.0%	45.8%	4.2%
Zaballa et al., 2012	18,304	Australia [Western metropolitan Sydney, New South Wales]		Women with singleton pregnancies	6.6%	6.2%	34.9%	3.4%
Enomoto et al., 2016	97,157	Japan	IADPSG/WHO '13	Women with singleton pregnancies included in the Japan Society of Obstetrics and Gynecology registry	3.6%	2.7%	69.4%	10.4%

Mayama et al., 2017	2,131	Japan		Pregnant women delivering at a hospital	0.6%			
Sugimura et al., 2020	6,066	Japan	IADPSG/WHO '13	Women with singleton pregnancies delivering at a hospital	1.9%	2.5%	63.9%	10.6%
Tanaka et al., 2021	36	Japan [Tokyo]	IADPSG/WHO '13	Pregnant women delivering at a hospital	36.4%		40%	
Sugiyama et al., 2017	1,730	Palau	Carpenter-Coustan/ACOG	Singleton pregnant women delivering at a hospital	3.1%	3.3%	26.3%	3.2%
Ha et al., 2019	1,666	Vietnam [Hanoi, Haiphong; Ho Chi Minh City]	IADPSG/WHO '13	Singleton pregnancy with no serious pre-existing health conditions.	21.1%	18.3%		
Hung & Hsieh, 2016	12,064	Taiwan	IADPSG/WHO '13	All singletons without fetal anomalies	8.7%	6.5%	76.4%	9.0%
Kim et al., 2022*	343	South Korea [Daegu]	Carpenter-Coustan/ACOG	Twin pregnancies	10.1%	9.1%	60.9%	6.5%
Kim et al., 2021	6,331	South Korea		Women with singleton pregnancies	4.5%	1.5%	53.8%	3.2%
Kim et al., 2020*	1,028	South Korea [Seoul]	Carpenter-Coustan/ACOG	Twin pregnancies	(7.5%)	4.7%	68.1%	8.8%
Kim et al., 2022	946	South Korea [Seoul]		Women with GDM delivering at a hospital			57.4%	9.1%
Lee et al., 2014	16,297	South Korea	Carpenter-Coustan/ACOG	Women with singleton live delivery	(3.5%)		(55.8%)	
Park et al., 2021*	4,348	South Korea		Women with twin deliveries	(9.9%)		(68.4%)	

Supplementary Table 2. Prevalence of GDM among women with BMI <25kg/m^2.

**Country**	**Average Prevalence**
Australia*	3.6% (1.2-6.6%)
Austria	4.4%
Brazil	7.5%
Canada	3.9%
Chile	1.4%
China*	17.6% (3.0-34.2%
Croatia	9.0% (3.3-14.7%)
Finland*	19.8% (5.4-34.2%)
France	8.6% (6.2-10.9%)
Hong Kong	6.4%
India	7.3% (0.0-24.4%)
Iran	5.8%
Ireland	10.4%
Israel	5.1%
Japan	10.6% (0.6-36.4%)
Jordan	0.6%
Mexico	10.8%
Nigeria	0.7%
Palau	3.0%
Pakistan	11.2%
Poland	19.3%
Saudi Arabia	5.4% (0.8-12.0%)
Singapore	8.4% (5.4-11.4%)
Slovenia	3.9% (2.0-5.8%)
South Korea	4.5%
Spain	6.6% (3.3-12.3%)
Sweden	0.1%
Taiwan	0.9%
Turkey	2.0% (0.9-3.1%)
United Kingdom	0.7% (0.1-1.0%)
United States†	5.0% (0.2-30.5%)
Vietnam	21.1%

*BMI range only extends up to <24 in some studies
†BMI range extends up to <26 in some studies

Supplementary Table 3. Prevalence of GDM among women with BMI <23kg/m^2.

Country	Average Prevalence
Bangladesh	18.7% (10.6-26.7%)
China*	18.0% (13.0-20.4%)
Croatia	12.7%
India	7.8% (2.8-12.7%)
Singapore	14.4%
South Korea	3.5% (3.5-9.9%)

*BMI range extends up to <23.9 in one study

Supplementary Table 4. Prevalence of GDM among women with BMI <18.5kg/m^2.

Country	Average Prevalence
Australia	3.4% (1.0-6.2%)
Bangladesh	10.4%
Canada	2.8%
Chile	0.9%
China	11.7% (1.4-18.2%)
Croatia	5.1% (2.78-7.4%)
Finland[†]	2.5% (1.2-3.8%)
France	3.5%
Hong Kong	4.9%
India*[†◊]	5.2% (0.0-13.6%)
Iran	0.0%
Israel	6.1%
Japan	2.6% (2.5-2.7%)
Jordan	0.1%
Nigeria	0.0%
Palau	3.3%
Pakistan	10.0%
Poland	15.8%
Saudi Arabia	4.5% (0.0-8.9%)
Singapore	5.1%
Slovenia	1.7% (1.3-2.1%)
South Korea	1.5%
Spain[◊]	8.7% (2.3-21.4%)
Sweden[†]	0.0%

Taiwan	6.5%
Turkey	1.3% (0.0-2.7%)
United Kingdom[†]	0.3% (0.0-0.6%)
United States*[†]	7.3% (0.0-73.6%)
Vietnam	18.3%

*BMI range extends up to <19.8 in some studies
†BMI range extends up to <20 in some studies
◊BMI range extends up to <20.9 in some studies

Supplementary Table 5. Prevalence of GDM among women with BMI <25kg/m^2 who delivered twins.

**Country**	**Average Prevalence**
Canada	5.1% (4.1-6.0%)
China*	20.8% (16.0-28.7%)
South Korea	9.2% (7.5-10.1%)

*BMI range only extends up to <24 in some studies

Supplementary Table 6. Prevalence of BMI <25kg/m^2 among women with GDM.

**Country**	**Average Prevalence**
Australia	25.8% (3.7-45.8%)
Brazil	22.2%
Canada	39.5% (39.0-40.0%)
Chile	49.8%
China*	70.5% (28.2-89.7%)
Croatia	55.0% (54.0-56.0%)
Denmark[†]	32.2%
Finland	20.5% (8.3-32.7%)
Hong Kong	65.6%
India	29.3% (0.6-60.0%)
Iran	32.4% (19.7-45.0%)
Italy[◊]	55.8%
Japan	53.3% (40.0-69.4%)
Jordan	29.6%
Nigeria	3.3%
Pakistan	13.2%
Palau	26.3%
Poland	35.5% (6.8-68.2%)
Portugal	43.2% (43.1-43.2%)

Saudi Arabia	14.4% (10.0-18.9%)
Scotland	16.9%
Singapore	43.9%
Slovenia	32.5% (4.3-48.2%)
South Korea	55.6% (53.8-57.4%)
Spain	43.3% (31.9-59.9%)
Sri Lanka†	77.8%
Sweden	80.6%
Taiwan	76.5%
Tunisia	28.8%
Turkey	51.3%
United Arab Emirates	8.3%
United Kingdom	17.7% (7.1-28.3%)
United States◊†*	41.8% (11.3-92.5%)
Qatar	25.7%

*BMI range extends up to <24 in some studies
◊BMI range extends up to <26 in some studies
†BMI range extends up to <27 in some studies
Germany and Austria have an aggregate prevalence of 26% [CITATION]

Supplementary Table 7. Prevalence of BMI <23kg/m^2 among women with GDM.

**Country**	**Average Prevalence**
Bangladesh	41.2% (10.0-72.4%)
China	61.7% (51.8-71.6%)
India	35.6% (16.3-35.6%)
South Korea	55.8%
Spain*	15.2%
Sri Lanka	27.3%

*BMI range only extends up to <20.9

Supplementary Table 8. Prevalence of BMI <18.5kg/m^2 among women with GDM.

**Country**	**Average Prevalence**
Australia	2.2% (0.4-4.2%)
Bangladesh	27.6%
Canada	2.8%
China	10.0% (3.5-17.4%)
Croatia	3.4% (3.3-3.4%)
Finland†	5.4% (5.0-5.9%)
Hong Kong	9.4%

India◊	5.3% (0.0-20.0%)
Iran	0.0%
Japan	10.5% (10.4-10.5%)
Jordan	0.4%
Nigeria	0.0%
Palau	3.2%
Poland	4.4% (0.6-10.9%)
Portugal	1.9%
Saudi Arabia	0.0%
Singapore	5.3%
Slovenia	1.5% (0.9-2.1%)
South Korea	6.2% (3.2-9.1%)
Spain	1.9% (1.7-2.2%)
Sri Lanka	3.0%
Sweden†	8.3%
Taiwan	9.0%
Tunisia	1.3%
Turkey	5.1%
United States*	13.5% (0.0-56.7%)

*BMI range extends up to <19.8 in some studies
◊BMI range extends up to <19.9 in some studies
†BMI range extends up to <20 in some studies

Supplementary Table 9. Prevalence of BMI <25kg/m^2 among women with GDM who delivered twins.

**Country**	**Average Prevalence**
China*◊	76.9% (72.5-85.9%)
Portugal	78.3%
South Korea*	65.8% (60.9-68.1%)

*BMI range only extends up to <23 in some studies
◊BMI range only extends up to <24 in some studies

Supplementary Figure 1.

(A) Prevalence of GDM among women with BMI <23kg/m^2.

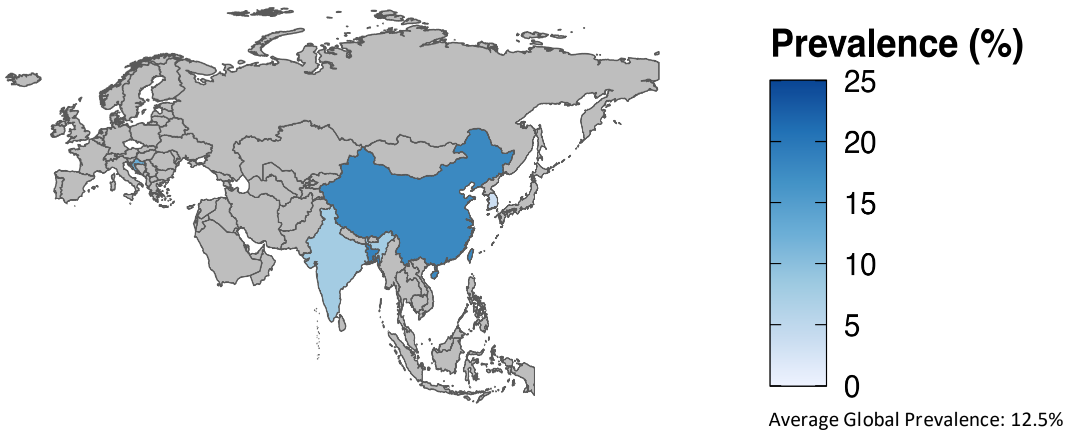

(B) Prevalence of GDM among women with BMI <25kg/m^2 who delivered twins.

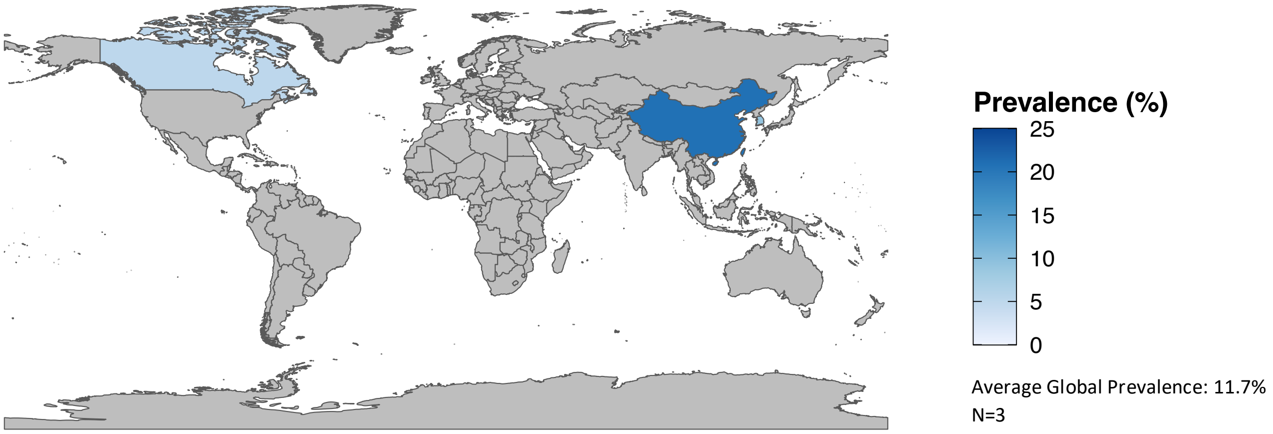

Supplementary Figure 2.

(A) Prevalence of BMI <23kg/m^2 among women with GDM.

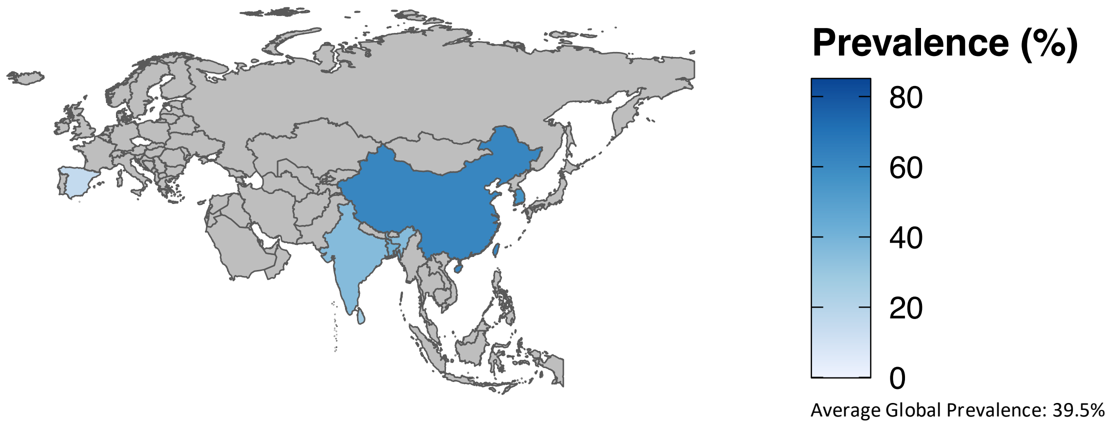

(B) Prevalence of BMI <25kg/m^2 among women with GDM who delivered twins.

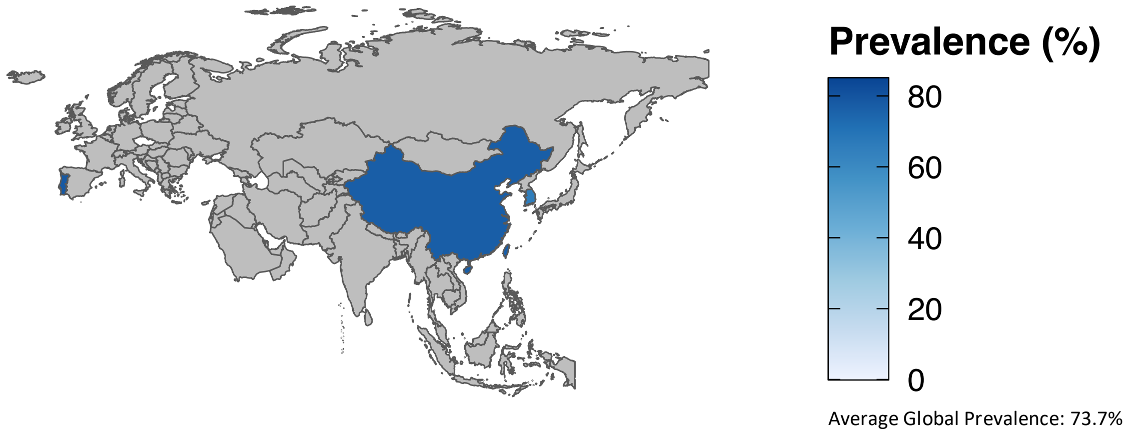

Additional Supplementary Material.

(A) Database search strategy

Ovid MEDLINE (ALL – 1946 to present)
Searched on June 12, 2023
No language, article type, or publication date limits

Line #	Search
1	Diabetes, Gestational/ and (lean or underweight or under-weight or non overweight or non obese or "not obese" or "without obesity").tw.
2	((lean or underweight or under-weight or non overweight or non obese or "not obese" or "without obesity") and (gestational diabet* or pregnancy induced diabet* or pregnan* diabet* or diabetes mellitus gravidarum or "diabetes in pregnancy" or "hyperglycemia in pregnancy" or maternal diabet*)).tw.
3	1 or 2

Ovid Embase (1974 to present)
Searched on June 12, 2023
No language, article type, or publication date limits

Line #	Search
1	pregnancy diabetes mellitus/ and (lean or underweight or under-weight or non overweight or non obese or "not obese" or "without obesity").tw.
2	((lean or underweight or under-weight or non overweight or non obese or "not obese" or "without obesity") and (gestational diabet* or pregnancy induced diabet* or pregnan* diabet* or diabetes mellitus gravidarum or "diabetes in pregnancy" or "hyperglycemia in pregnancy" or maternal diabet*)).tw.
3	1 or 2

Cochrane Library (Wiley)
Searched on June 12, 2023
No language, article type, or publication date limits

ID	Search

#1	(("lean" OR "underweight" OR "under-weight" OR "non overweight" OR "non obese" OR "not obese" OR "without obesity") AND ("gestational diabetes" OR "pregnancy induced diabet" OR "pregnant diabetes" OR "diabetes mellitus gravidarum" OR "diabetes in pregnancy" or "hyperglycemia in pregnancy" or maternal diabet*)):ti,ab

(B) Reference list of included studies.

1. Abadia S, Sebai I, Raddedi S, Abdesslem H, Salem A, Zemni Z, et al. Impact of obesity on gestational diabetes mellitus. Obesity Facts. 2021;14(SUPPL 1):44-5.
2. Ahmed SR, Ellah MAA, Mohamed OA, Eid HM. Prepregnancy obesity and pregnancy outcome. International journal of health sciences. 2009;3(2):203-8.
3. Akgol S, Rapisarda AMC, Budak MS, Caruso S, Cianci A, Reyes-Munoz E, et al. The effect of obesity on the onset of spontaneous labor and scheduled delivery rates in term pregnancies. Taiwanese journal of obstetrics & gynecology. 2020;59(1):34-8.
4. Akinyemi OA, Tanna R, Adetokunbo S, Omokhodion O, Fasokun M, Akingbule AS, et al. Increasing Pre-pregnancy Body Mass Index and Pregnancy Outcomes in the United States. Cureus. 2022;14(9):e28695.
5. Al-Obaidly S, Parrish J, Murphy KE, Maxwell C. Maternal pre-gravid body mass index and obstetric outcomes in twin gestations. Journal of perinatology : official journal of the California Perinatal Association. 2014;34(6):425-8.
6. Alvarez Cuenod JS, Sanchez Sanchez V, Gonzalez Martin JM, Emergui Zrihen Y, Suarez Guillen V, Ribary Domingo A, et al. Extreme values of maternal BMI: Determinants of worse obstetric and perinatal outcomes. Clinica e Investigacion en Ginecologia y Obstetricia. 2022;49(3):100754.
7. Amini D, Driggers R, Umans JG, Singh J, Miodovnik M. Overweight or obese: The impact of Body Mass Index (BMI) on rates of gestational diabetes. Reproductive Sciences. 2010;17(3 SUPPL. 1):186A.
8. Anand S, Rana S, Maiti S. Implications of extremes of body weight on pregnancy outcome. Archives of Disease in Childhood: Fetal and Neonatal Edition. 2011;96(SUPPL. 1):Fa112.
9. Anchala B, Ruchi R. Impact of Pre-pregnancy Body Mass Index on Neonatal Outcome. Iranian Journal of Neonatology. 2021;12(4):77-84.
10. Asad U, Khan S, Zulfiqar S, Munawar R, Akhtar S, Khalid Z. Maternal and Fetal Outcomes in Obesity Complicated Pregnancies. Pakistan Journal of Medical and Health Sciences. 2022;16(10):518-21.
11. Avci ME, Sanlikan F, Celik M, Avci A, Kocaer M, Gocmen A. Effects of maternal obesity on antenatal, perinatal and neonatal outcomes. The journal of maternal-fetal & neonatal medicine : the official journal of the European Association of Perinatal Medicine, the Federation of Asia and Oceania Perinatal Societies, the International Society of Perinatal Obstetricians. 2015;28(17):2080-3.

12. Baeten JM, Bukusi EA, Lambe M. Pregnancy complications and outcomes among overweight and obese nulliparous women. American journal of public health. 2001;91(3):436-40.
13. Bhowmik B, Siddique T, Majumder A, Mdala I, Hossain IA, Hassan Z, et al. Maternal BMI and nutritional status in early pregnancy and its impact on neonatal outcomes at birth in Bangladesh. BMC pregnancy and childbirth. 2019;19(1):413.
14. Blackwell SC. Are adverse perinatal outcomes in twin pregnancies increased with maternal obesity? Reproductive Sciences. 2012;19(3 SUPPL. 1):131A.
15. Cai S, Aris IM, Yuan WL, Tan KH, Godfrey KM, Gluckman PD, et al. Neonatal amygdala microstructure mediates the relationship between gestational glycemia and offspring adiposity. BMJ open diabetes research & care. 2021;9(1).
16. Carducci Artenisio A, Corrado F, Sobbrio G, Bruno L, Todisco L, Galletta MG, et al. Glucose tolerance and insulin secretion in pregnancy. Diabetes, nutrition & metabolism. 1999;12(4):264-70.
17. Catalano PM, Roman NM, Tyzbir ED, Merritt AO, Driscoll P, Amini SB. Weight gain in women with gestational diabetes. Obstetrics and gynecology. 1993;81(4):523-8.
18. Chaurasia AK, Dwedi LS. A Study to Determine the Early Pregnancy BMI, the Prevalence of Various Levels of BMI, and the Correlation in Order to Analyse the Influence of Low Weight, Overweight, and Obesity on Mother and Foetal Outcomes. International Journal of Pharmaceutical and Clinical Research. 2021;13(3):424-32.
19. Chen X, Scholl TO, Leskiw M, Savaille J, Stein TP. Differences in maternal circulating fatty acid composition and dietary fat intake in women with gestational diabetes mellitus or mild gestational hyperglycemia. Diabetes care. 2010;33(9):2049-54.
20. Chen Xu J, Coelho A. Association between Body Mass Index and Gestational Weight Gain with Obstetric and Neonatal Complications in Pregnant Women with Gestational Diabetes. Acta medica portuguesa. 2022;35(10):718-28.
21. Cheney C, Shragg P, Hollingsworth D. Demonstration of heterogeneity in gestational diabetes by a 400-kcal breakfast meal tolerance test. Obstetrics and gynecology. 1985;65(1):17-23.
22. Chodick G, Omer-Gilon M, Derazne E, Puris G, Rotem R, Tzur D, et al. Adolescent body mass index and changes in pre-pregnancy body mass index in relation to risk of gestational diabetes. EClinicalMedicine. 2021;42(101733727):101211.
23. Chung HK. Association of pre-pregnancy body mass index and gestational weight gain with pregnancy outcomes. Hong Kong Journal of Gynaecology Obstetrics and Midwifery. 2022;22(2):66-72.
24. Cunningham CE, Teale GR. A profile of body mass index in a large rural Victorian obstetric cohort. The Medical journal of Australia. 2013;198(1):39-42.
25. De A, Nigam A, Sharma S, Anwar A. Comparison of Feto-maternal Outcomes Among Various BMI Groups As Per Asia Pacific Standards: An Observational Retrospective Comparative Study in a Private Tertiary Care Center in Delhi. Journal of Obstetrics and Gynecology of India. 2023((De, Nigam, Sharma, Anwar) Department of Obstetrics and Gynecology, Hamdard Institute of Medical Sciences and Research (HIMSR), Jamia Hamdard, New Delhi 110062, India).

26. Delmis J, Pavic M, Ivanisevic M, Juras J, Herman M, Oreskovic S. Body mass index and pregnancy outcome. Gynaecologia et Perinatologia. 2015;24(3):99-105.
27. Denison FC, Norwood P, Bhattacharya S, Duffy A, Mahmood T, Morris C, et al. Association between maternal body mass index during pregnancy, short-term morbidity, and increased health service costs: a population-based study. BJOG : an international journal of obstetrics and gynaecology. 2014;121(1):72-82.
28. Ducarme G, Rodrigues A, Aissaoui F, Davitian C, Pharisien I, Uzan M. [Pregnancy in obese patients: which risks is it necessary to fear?]. Gynecologie, obstetrique & fertilite. 2007;35(1):19-24.
29. Ehrenberg HM, Durnwald CP, Catalano P, Mercer BM. The influence of obesity and diabetes on the risk of cesarean delivery. American journal of obstetrics and gynecology. 2004;191(3):969-74.
30. Ekin A, Gezer C, Taner CE, Solmaz U, Ozeren M. Effect of body mass index before pregnancy on perinatal outcomes. Medical Journal of Bakirkoy. 2017;13(1):20-5.
31. Enomoto K, Aoki S, Toma R, Fujiwara K, Sakamaki K, Hirahara F. Pregnancy Outcomes Based on Pre-Pregnancy Body Mass Index in Japanese Women. PloS one. 2016;11(6):e0157081.
32. Fakhrul-Alam M, Sharmin J, Mashfiqul H, Nusrat S, Mohona Z, Rakibul-Hasan M, et al. Insulin secretory defect may be the major determinant of GDM in lean mothers. Journal of Clinical and Translational Endocrinology. 2020;20((Fakhrul-Alam, Sharmin-Jahan, Mashfiqul-Hasan, Nusrat-Sultana, Mohona-Zaman, Rakibul-Hasan, Farid-Uddin, Hasanat) Department of Endocrinology, Bangabandhu Sheikh Mujib Medical University, Bangladesh):100226.
33. Farooq S, Baloch S, Awan S, Fakharunissa. Influence of Body Mass Index in Pregnancy on Maternal and Fetal Outcome. Pakistan Journal of Medical and Health Sciences. 2022;16(5):616-9.
34. Feng N, Huang X. Effect of pre-pregnancy body mass index and gestational weight gain on perinatal outcomes. International Journal of Clinical and Experimental Medicine. 2021;14(8):2180-8.
35. Feresu SA, Wang Y, Dickinson S. Relationship between maternal obesity and prenatal, metabolic syndrome, obstetrical and perinatal complications of pregnancy in Indiana, 2008-2010. BMC pregnancy and childbirth. 2015;15(100967799):266.
36. Foo XY, Greer RM, Kumar S. Impact of Maternal Body Mass Index on Intrapartum and Neonatal Outcomes in Brisbane, Australia, 2007 to 2013. Birth (Berkeley, Calif). 2016;43(4):358-65.
37. Gao L, Lei C, Zhou S, Liao Q, Mei L, Zhong Q, et al. Investigation of optimal gestational weight gain for twin pregnancy in Southwest China: a retrospective study. Scientific reports. 2023;13(1):5059.
38. Garmendia ML, Matus O, Mondschein S, Kusanovic JP. Gestational weight gain recommendations for Chilean women: a mathematical optimization approach. Public health. 2018;163(qi7, 0376507):80-6.
39. Gu C, Wu W, Lai K, Li H, Wu L, Lu W, et al. Maternal pre-pregnancy BMI, MTHFR polymorphisms, and the risk of adverse pregnancy outcomes in pregnant women from South China: a retrospective cohort study. BMC pregnancy and childbirth. 2023;23(1):295.

40. Ha AVV, Zhao Y, Pham NM, Nguyen CL, Nguyen PTH, Chu TK, et al. Postpartum weight retention in relation to gestational weight gain and pre-pregnancy body mass index: A prospective cohort study in Vietnam. Obesity Research and Clinical Practice. 2019;13(2):143-9.
41. Hantoushzadeh S, Sheikh M, Bosaghzadeh Z, Ghotbizadeh F, Tarafdari A, Panahi Z, Shariat M. The impact of gestational weight gain in different trimesters of pregnancy on glucose challenge test and gestational diabetes. Postgraduate medical journal. 2016;92(1091):520-4.
42. Hashemipour S, Haji Seidjavadi E, Maleki F, Esmailzadehha N, Movahed F, Yazdi Z. Level of maternal triglycerides is a predictor of fetal macrosomia in non-obese pregnant women with gestational diabetes mellitus. Pediatrics and neonatology. 2018;59(6):567-72.
43. He M, Curran P, Martin S, Lambert-Messerlian G, Bourjeily G. Placental findings in pregnancies with maternal obesity. Placenta. 2012;33(9):A120.
44. He S, Allen JC, Razali NS, Chern BSM, Tan KH. Association between gestational weight gain and pregnancy outcomes in a Singaporean population: A prospective cohort study. European Journal of Obstetrics and Gynecology and Reproductive Biology. 2022;272((He, Razali, Chern) Division of Obstetrics and Gynaecology, KK Women's and Children's Hospital, 100 Bukit Timah Road, Singapore 229899, Singapore(Allen) Centre for Quantitative Medicine, Duke-NUS Medical School, 8 College Road, Singapore 169857, Singapore):160-5.
45. Hollingsworth DR, Vaucher Y, Yamamoto TR. Diabetes in pregnancy in Mexican Americans. Diabetes care. 1991;14(7):695-705.
46. Holopainen LS, Tahtinen HH, Gissler M, Korhonen PE, Ekblad MO. Pre-pregnancy body surface area and risk for gestational diabetes mellitus. Acta diabetologica. 2023;60(4):527-34.
47. Houser M, Tuuli M, Macones G, Odibo A. Is the association between gestational diabetes and preeclampsia modified by obesity? American Journal of Obstetrics and Gynecology. 2011;204(1 SUPPL.):S115.
48. Hu J, Liu Y, Wei X, Li L, Gao M, Liu Y, et al. Association of gestational diabetes mellitus with offspring weight status across infancy: a prospective birth cohort study in China. BMC pregnancy and childbirth. 2021;21(1):21.
49. Hung T-H, Hsieh Ts-Ta. Pregestational body mass index, gestational weight gain, and risks for adverse pregnancy outcomes among Taiwanese women: A retrospective cohort study. Taiwanese journal of obstetrics & gynecology. 2016;55(4):575-81.
50. Jahan E, Balouch I, Memon SM, Naveel T, Fakharunissa, Jabeen A. Frequency of Maternal Complications in Obese and Non Obese Pregnant Patients. Pakistan Journal of Medical and Health Sciences. 2022;16(4):506-8.
51. Jia X, Li N, Gao S, Ye R, Wang J, Liu X, Li Z. The impact of self-reported preconception body mass index on gestational abnormal glucose tolerance in a Chinese center. Journal of diabetes and its complications. 2018;32(10):951-4.
52. Karasneh RA, Migdady FH, Alzoubi KH, Al-Azzam SI, Khader YS, Nusair MB. Trends in maternal characteristics, and maternal and neonatal outcomes of women with gestational diabetes: A study from Jordan. Annals of medicine and surgery (2012). 2021;67(101616869):102469.

53. Khalak R, Cummings J, Dexter S. Maternal obesity: significance on the preterm neonate. International journal of obesity (2005). 2015;39(10):1433-6.
54. Kim M, Hur K-Y, Choi S-J, Oh S-Y, Roh C-R. Influence of Pre-Pregnancy Underweight Body Mass Index on Fetal Abdominal Circumference, Estimated Weight, and Pregnancy Outcomes in Gestational Diabetes Mellitus. Diabetes & metabolism journal. 2022;46(3):499-505.
55. Kim MJ, Kim HM, Cha H-H, Seong WJ. Correlation between Maternal Weight Gain in Each Trimester and Fetal Growth According to Pre-Pregnancy Maternal Body Mass Index in Twin Pregnancies. Medicina (Kaunas, Lithuania). 2022;58(9).
56. Kim S-Y, Hong S-Y, Kim Y, Kwon DY, Park H, Sung J-H, et al. Maternal pre-pregnancy body mass index and the risk for gestational diabetes mellitus in women with twin pregnancy in South Korea. Taiwanese journal of obstetrics & gynecology. 2021;60(5):863-8.
57. Kim S-Y, Oh S-Y, Sung J-H, Choi S-J, Roh C-R, Lee SM, et al. Validation of a Strict Obesity Definition Proposed for Asians to Predict Adverse Pregnancy Outcomes in Korean Pregnant Women. Journal of Korean medical science. 2021;36(44):e281.
58. Kleinwechter HJ, Weber KS, Mingers N, Ramsauer B, Schaefer-Graf UM, Groten T, et al. Gestational diabetes mellitus and COVID-19: results from the COVID-19-Related Obstetric and Neonatal Outcome Study (CRONOS). American journal of obstetrics and gynecology. 2022;227(4):631.e1-.e19.
59. Knight-Agarwal CR, Williams LT, Davis D, Davey R, Cochrane T, Zhang H, Rickwood P. Association of BMI and interpregnancy BMI change with birth outcomes in an Australian obstetric population: a retrospective cohort study. BMJ open. 2016;6(5):e010667.
60. Kumari AS. Pregnancy outcome in women with morbid obesity. International journal of gynaecology and obstetrics: the official organ of the International Federation of Gynaecology and Obstetrics. 2001;73(2):101-7.
61. Kutchi I, Chellammal P, Akila A. Maternal Obesity and Pregnancy Outcome: in Perspective of New Asian Indian Guidelines. Journal of obstetrics and gynaecology of India. 2020;70(2):138-44.
62. Lan X, Zhang Y-Q, Dong H-L, Zhang J, Zhou F-M, Bao Y-H, et al. Excessive gestational weight gain in the first trimester is associated with risk of gestational diabetes mellitus: a prospective study from Southwest China. Public health nutrition. 2020;23(3):394-401.
63. Lee JM, Kim MJ, Kim MY, Han JY, Ahn HK, Choi JS, et al. Gestational weight gain is an important risk factor for excessive fetal growth. Obstetrics & gynecology science. 2014;57(6):442-7.
64. Lewandowska M. Gestational Diabetes Mellitus (GDM) Risk for Declared Family History of Diabetes, in Combination with BMI Categories. International journal of environmental research and public health. 2021;18(13).
65. Li G, Kong L, Zhang L, Fan L, Su Y, Rose JC, Zhang W. Early Pregnancy Maternal Lipid Profiles and the Risk of Gestational Diabetes Mellitus Stratified for Body Mass Index. Reproductive sciences (Thousand Oaks, Calif). 2015;22(6):712-7.
66. Li M-Y, Rawal S, Hinkle SN, Zhu Y-Y, Tekola-Ayele F, Tsai MY, et al. Sex Hormone-binding Globulin, Cardiometabolic Biomarkers, and Gestational Diabetes:

A Longitudinal Study and Meta-analysis. Maternal-fetal medicine (Wolters Kluwer Health, Inc). 2020;2(1):2-9.
67. Lin D, Chen G, Fan D, Li P, Ma H, Wu S, et al. The gestational weight gain and perinatal outcomes among underweight women with twin pregnancies: Propensity score matched analysis from a three-year retrospective cohort. European journal of obstetrics, gynecology, and reproductive biology. 2019;243(e4l, 0375672):97-102.
68. Lindholm ES, Altman D, Norman M, Blomberg M. Health Care Consumption during Pregnancy in relation to Maternal Body Mass Index: A Swedish Population Based Observational Study. Journal of obesity. 2015;2015(101526295):215683.
69. Lipworth H, Melamed N, Berger H, Geary M, McDonald SD, Murray-Davis B, et al. Maternal weight gain and pregnancy outcomes in twin gestations. American journal of obstetrics and gynecology. 2021;225(5):532.e1-.e12.
70. Liu L, Hong Z, Zhang L. Associations of prepregnancy body mass index and gestational weight gain with pregnancy outcomes in nulliparous women delivering single live babies. Scientific reports. 2015;5(101563288):12863.
71. Liu X, Du J, Wang G, Chen Z, Wang W, Xi Q. Effect of pre-pregnancy body mass index on adverse pregnancy outcome in north of China. Archives of gynecology and obstetrics. 2011;283(1):65-70.
72. Lowry J, Lake-Burger H, Salemi J, Tanner JP, Kirby R. Proportion of critical congenital heart defects attributable to unhealthy prepregnancy body mass index among women with live births in Florida, 2004-2013. Birth Defects Research. 2018;110(9):804.
73. Loy SL, Cheng TS, Colega MT, Cheung YB, Godfrey KM, Gluckman PD, et al. Predominantly night-time feeding and maternal glycaemic levels during pregnancy. The British journal of nutrition. 2016;115(9):1563-70.
74. Lucovnik M, Blickstein I, Mirkovic T, Verdenik I, Bricelj K, Vidmar Simic M, et al. Effect of pre-gravid body mass index on outcomes of pregnancies following in vitro fertilization. Journal of assisted reproduction and genetics. 2018;35(7):1309-15.
75. Lucovnik M, Blickstein I, Steblovnik L, Verdenik I, Bregar AT, Tul N. Gestational weight gain according to the 2009 Institute of Medicine guidelines in patients with gestational diabetes. American Journal of Obstetrics and Gynecology. 2014;210(1 SUPPL. 1):S195.
76. Lyu J, Sun Y, Ji Y, Liu N, Zhang S, Lin H, et al. Optimal Gestational Weight Gain for Women with Gestational Diabetes Mellitus - China, 2011-2021. China CDC weekly. 2023;5(9):189-93.
77. Machado C, Monteiro S, Oliveira MJ. Impact of overweight and obesity on pregnancy outcomes in women with gestational diabetes - results from a retrospective multicenter study. Archives of endocrinology and metabolism. 2020;64(1):45-51.
78. Mackeen AD, Angras K, Muchisky A, Young A. 811: Delineation of risks associated with specific pre-pregnancy BMI classes. American Journal of Obstetrics and Gynecology. 2019;220(1 Supplement):S531-S2.
79. Madhavan A, Beena Kumari R, Sanal MG. A pilot study on the usefulness of body mass index and waist hip ratio as a predictive tool for gestational diabetes in Asian Indians. Gynecological endocrinology : the official journal of the International Society of Gynecological Endocrinology. 2008;24(12):701-7.

80. Madhavi K, Chandra GK, Afsha A. A STUDY ON THE EFFECT OF OBESITY ON PREGNANCY OUTCOME. European Journal of Molecular and Clinical Medicine. 2022;9(4):3083-98.
81. Mandal D, Manda S, Rakshi A, Dey RP, Biswas SC, Banerjee A. Maternal obesity and pregnancy outcome: a prospective analysis. The Journal of the Association of Physicians of India. 2011;59(hg7, 7505585):486-9.
82. Martinez-Frias ML, Frias JP, Bermejo E, Rodriguez-Pinilla E, Prieto L, Frias JL. Pre-gestational maternal body mass index predicts an increased risk of congenital malformations in infants of mothers with gestational diabetes. Diabetic medicine : a journal of the British Diabetic Association. 2005;22(6):775-81.
83. Mayama M, Nomura E, Yamada T, Takeda T, Uno K, Tano S, et al. The effect of pre-pregnancy body mass index on perinatal outcomes in normal weight women. Journal of Perinatal Medicine. 2017;45(Supplement 2):554.
84. McGoldrick A, O'Higgins A, O'Dwyer V, O'Connor C, Farah N, Turner M. Risk of gestational diabetes mellitus analysed by the rate of weight gain before screening. American Journal of Obstetrics and Gynecology. 2013;208(1 SUPPL.1):S112.
85. McIntyre HD, Gibbons KS, Flenady VJ, Callaway LK. Overweight and obesity in Australian mothers: epidemic or endemic? The Medical journal of Australia. 2012;196(3):184-8.
86. Meher Un N, Aslam M, Ahmed SR, Rajab M, Kattea L. Impact of obesity on fetomaternal outcome in pregnant saudi females. International journal of health sciences. 2009;3(2):187-95.
87. Min Y, Ghebremeskel K, Lowy C, Thomas B, Crawford MA. Adverse effect of obesity on red cell membrane arachidonic and docosahexaenoic acids in gestational diabetes. Diabetologia. 2004;47(1):75-81.
88. Naik R, Karmali D, Nagarsenkar A, Mainath S, Pednekar G. Effect of Pre-pregnancy Maternal Body Mass Index on Obstetric Outcomes in a Tertiary Care Hospital in Goa, India. Journal of Obstetrics and Gynecology of India. 2022;72(2):141-6.
89. Ogonowski J, Miazgowski T, Kuczynska M, Krzyzanowska-Swiniarska B, Celewicz Z. Pregravid body mass index as a predictor of gestational diabetes mellitus. Diabetic medicine : a journal of the British Diabetic Association. 2009;26(4):334-8.
90. Ogunyemi D, Hullett S, Leeper J, Risk A. Prepregnancy body mass index, weight gain during pregnancy, and perinatal outcome in a rural black population. The Journal of maternal-fetal medicine. 1998;7(4):190-3.
91. Olmos PR, Borzone GR, Olmos RI, Valencia CN, Bravo FA, Hodgson MI, et al. Gestational diabetes and pre-pregnancy overweight: possible factors involved in newborn macrosomia. The journal of obstetrics and gynaecology research. 2012;38(1):208-14.
92. Park J-Y, Kim WJ, Chung YH, Kim B, Park Y, Park IY, Ko HS. Association between pregravid liver enzyme levels and gestational diabetes in twin pregnancies: a secondary analysis of national cohort study. Scientific reports. 2021;11(1):18695.
93. Pathi A, Esen U, Hildreth A. A comparison of complications of pregnancy and delivery in morbidly obese and non-obese women. Journal of obstetrics and gynaecology : the journal of the Institute of Obstetrics and Gynaecology. 2006;26(6):527-30.

94. Perea V, Simo-Servat A, Quiros C, Alonso-Carril N, Valverde M, Urquizu X, et al. Role of Excessive Weight Gain During Gestation in the Risk of ADHD in Offspring of Women With Gestational Diabetes. The Journal of clinical endocrinology and metabolism. 2022;107(10):e4203-e11.
95. Ravnsborg T, Andersen LLT, Trabjerg ND, Rasmussen LM, Jensen DM, Overgaard M. First-trimester multimarker prediction of gestational diabetes mellitus using targeted mass spectrometry. Diabetologia. 2016;59(5):970-9.
96. Relph S, Guo Y, Harvey ALJ, Vieira MC, Corsi DJ, Gaudet LM, Pasupathy D. Characteristics associated with uncomplicated pregnancies in women with obesity: a population-based cohort study. BMC pregnancy and childbirth. 2021;21(1):182.
97. Rocha AdS, Bernardi JR, Matos S, Kretzer DC, Schoffel AC, Goldani MZ, de Azevedo Magalhaes JA. Maternal visceral adipose tissue during the first half of pregnancy predicts gestational diabetes at the time of delivery - a cohort study. PloS one. 2020;15(4):e0232155.
98. Romero Gutierrez G, Urbina Ortiz FJ, Ponce de Leon ALP, Amador N. [Maternal and fetal morbidity in obese pregnant women]. Ginecologia y obstetricia de Mexico. 2006;74(9):483-7.
99. Rudra CB, Sorensen TK, Leisenring WM, Dashow E, Williams MA. Weight characteristics and height in relation to risk of gestational diabetes mellitus. American journal of epidemiology. 2007;165(3):302-8.
100. Sahu MT, Agarwal A, Das V, Pandey A. Impact of maternal body mass index on obstetric outcome. The journal of obstetrics and gynaecology research. 2007;33(5):655-9.
101. Salmon C, Thibon P, Prime L, Renouf S, Dreyfus M, Dolley P. Impact of maternal underweight on obstetric and neonatal prognosis: A retrospective study. European Journal of Obstetrics and Gynecology and Reproductive Biology. 2021;260((Salmon) Service de Gynecologie-Obstetrique, CHU de Caen Normandie, Normandie Univ, UNICAEN, Caen 14000, France(Thibon, Prime) Reseau Perinatal, Centre Hospitalo-Universitaire, Caen F-14033, France(Renouf, Dreyfus, Dolley) Service de Gynecologie-Obstetriq):6-9.
102. Schaefer KK, Xiao W, Chen Q, He J, Lu J, Chan F, et al. Prediction of gestational diabetes mellitus in the Born in Guangzhou Cohort Study, China. International journal of gynaecology and obstetrics: the official organ of the International Federation of Gynaecology and Obstetrics. 2018;143(2):164-71.
103. Schuster M, Neubert A, Kirchner L, Paglia M. The impact of body mass index on pregnancy complications. American Journal of Obstetrics and Gynecology. 2015;212(1 SUPPL. 1):S421.
104. Sebire NJ, Jolly M, Harris J, Regan L, Robinson S. Is maternal underweight really a risk factor for adverse pregnancy outcome? A population-based study in London. BJOG : an international journal of obstetrics and gynaecology. 2001;108(1):61-6.
105. Senbanjo OC, Akinlusi FM, Ottun TA. Early pregnancy body mass index, gestational weight gain and perinatal outcome in an obstetric population in Lagos, Nigeria. The Pan African medical journal. 2021;39(101517926):136.
106. Shin D, Song WO. Prepregnancy body mass index is an independent risk factor for gestational hypertension, gestational diabetes, preterm labor, and small- and large-for-gestational-age infants. The journal of maternal-fetal & neonatal medicine : the

official journal of the European Association of Perinatal Medicine, the Federation of Asia and Oceania Perinatal Societies, the International Society of Perinatal Obstetricians. 2015;28(14):1679-86.

107. Simoes T, Queiros A, Valdoleiros S, Marujo AT, Felix N, Blickstein I. Concurrence of gestational diabetes and pre-gravid obesity ("diabesity") in twin gestations. The journal of maternal-fetal & neonatal medicine : the official journal of the European Association of Perinatal Medicine, the Federation of Asia and Oceania Perinatal Societies, the International Society of Perinatal Obstetricians. 2017;30(15):1813-5.

108. Somani S, Misra M, Tahilramani H, Kamlesh Kumari K. Effect of Maternal Body Mass Index on Pregnancy Outcome. International Journal of Pharmaceutical and Clinical Research. 2022;14(11):265-73.

109. Song L, Wang N, Peng Y, Sun B, Cui W. Placental lipid transport and content in response to maternal overweight and gestational diabetes mellitus in human term placenta. Nutrition, metabolism, and cardiovascular diseases : NMCD. 2022;32(3):692-702.

110. Sugimura R, Kohmura-Kobayashi Y, Narumi M, Furuta-Isomura N, Oda T, Tamura N, et al. Comparison of three classification systems of Prepregnancy Body Mass Index with Perinatal Outcomes in Japanese Obese Pregnant Women: A retrospective study at a single center. International journal of medical sciences. 2020;17(13):2002-12.

111. Sugiyama MS, Cash HL, Roseveare C, Reklai R, Basilius K, Madraisau S. Assessment of Gestational Diabetes and Associated Risk Factors and Outcomes in the Pacific Island Nation of Palau. Maternal and child health journal. 2017;21(10):1961-6.

112. Sun D, Li F, Zhang Y, Xu X. Associations of the pre-pregnancy BMI and gestational BMI gain with pregnancy outcomes in Chinese women with gestational diabetes mellitus. International journal of clinical and experimental medicine. 2014;7(12):5784-9.

113. Tanaka K, Harata G, Miyazawa K, He F, Tanigaki S, Kobayashi Y. The gut microbiota of non-obese Japanese pregnant women with gestational diabetes mellitus. Bioscience of microbiota, food and health. 2022;41(1):4-11.

114. Tang F, Guan L, Liu X, Fan P, Zhou M, Wu Y, et al. A Common R219K Variant of ATP-Binding Cassette Transporter A1 Gene Alters Atherometabolic Traits in Pregnant Women With Gestational Diabetes Mellitus. Frontiers in endocrinology. 2021;12(101555782):782453.

115. Teshome AA, Li Q, Garoma W, Chen X, Wu M, Zhang Y, et al. Gestational diabetes mellitus, pre-pregnancy body mass index and gestational weight gain predicts fetal growth and neonatal outcomes. Clinical nutrition ESPEN. 2021;42(101654592):307-12.

116. Thayer SM, Owens S, Garg B, Caughey AB. 695 Cesarean delivery incidence by maternal BMI and diabetes status in multiparous women without prior cesarean. American Journal of Obstetrics and Gynecology. 2021;224(2 Supplement):S435-S6.

117. Tripathi G, Chourey N, Rn H, Sandhu N, Sinha P, Patel P, Vaswani P. "Effect of body mass index on pregnancy outcome" - A prospective study. Asian Journal of Pharmaceutical and Clinical Research. 2021;14(11):60-4.

118. Trivikrama HS, Krishnan R, Chellamma VK. DOES MATERNAL PRE-PREGNANCY UNDERWEIGHT AFFECT PREGNANCY OUTCOMES IN SINGLETON PREGNANCIES? OUR EXPERIENCE AT A TERTIARY CARE CENTER IN NORTH KERALA. Asian Journal of Pharmaceutical and Clinical Research. 2023;16(4):93-6.
119. Trojner Bregar A, Blickstein I, Brzan Simenc G, Jansa V, Verdenik I, Lucovnik M, Tul N. Perinatal Advantages and Disadvantages of Being Underweight before Pregnancy: A Population-Based Study. Gynecologic and obstetric investigation. 2017;82(3):303-6.
120. Verma A, Shrimali L. Maternal body mass index and pregnancy outcome. Journal of clinical and diagnostic research : JCDR. 2012;6(9):1531-3.
121. Vidanalage CJK, Senarth U, Silva KD, Lekamge U, Liyanage IJ. Effects of initial body mass index on development of gestational diabetes in a rural Sri Lankan population: A case-control study. Diabetes & metabolic syndrome. 2016;10(2 Suppl 1):S110-3.
122. Vigneault J, Lemieux S, Garneau V, Weisnagel SJ, Tchernof A, Robitaille J. Association between metabolic deteriorations and prior gestational diabetes according to weight status. Obesity (Silver Spring, Md). 2015;23(2):345-50.
123. Vince K, Brkic M, Poljicanin T, Matijevic R. Prevalence and impact of pre-pregnancy body mass index on pregnancy outcome: a cross-sectional study in Croatia. Journal of obstetrics and gynaecology : the journal of the Institute of Obstetrics and Gynaecology. 2021;41(1):55-9.
124. Vivian Ukah U, Bayrampour H, Sabr Y, Razaz N, Chan WS, Lim KI, Lisonkova S. Association between gestational weight gain and severe adverse birth outcomes in Washington State, US: A population-based retrospective cohort study, 2004-2013. PLoS Medicine. 2019;16(12):e1003009.
125. Wahabi H, Esmaeil S, Fayed A. Maternal Prepregnancy Weight and Pregnancy Outcomes in Saudi Women: Subgroup Analysis from Riyadh Mother and Baby Cohort Study (RAHMA). BioMed research international. 2021;2021(101600173):6655942.
126. Wang C, Lin L, Su R, Zhu W, Wei Y, Yan J, et al. Hemoglobin levels during the first trimester of pregnancy are associated with the risk of gestational diabetes mellitus, pre-eclampsia and preterm birth in Chinese women: a retrospective study. BMC pregnancy and childbirth. 2018;18(1):263.
127. Wang D, Ding W, Ding C, Chen H, Zhao W, Sun B, Wang Z. Higher Peripheral Cholesterol and a Positive Correlation With Risk for Large-For-Gestational-Age Neonates in Pre-Pregnancy Underweight Women. Frontiers in Endocrinology. 2021;12((Wang, Ding, Ding, Chen, Wang) Department of Obstetrics and Gynecology, The First Affiliated Hospital of Sun Yat-sen University, Guangzhou, China(Zhao, Sun) Department of Obstetrics and Gynecology, Shenzhen Second People's Hospital, Shenzhen, China):760934.
128. Wang F, Liang ZX, Mao WR, He SN, Chen DQ. Influence of pre-pregnancy body mass index and gestational weight gain in twin pregnancies on blood glucose, serum lipid and perinatal outcomes. Clinical and Experimental Obstetrics and Gynecology. 2020;47(3):376-82.

129. Wang N, Ding Y, Wu J. Effects of pre-pregnancy body mass index and gestational weight gain on neonatal birth weight in women with gestational diabetes mellitus. Early human development. 2018;124(edh, 7708381):17-21.
130. Wei Y-M, Liu X-Y, Shou C, Liu X-H, Meng W-Y, Wang Z-L, et al. Value of fasting plasma glucose to screen gestational diabetes mellitus before the 24th gestational week in women with different pre-pregnancy body mass index. Chinese medical journal. 2019;132(8):883-8.
131. Wei Y-M, Yang H-X, Zhu W-W, Liu X-Y, Meng W-Y, Wang Y-Q, et al. Risk of adverse pregnancy outcomes stratified for pre-pregnancy body mass index. The journal of maternal-fetal & neonatal medicine : the official journal of the European Association of Perinatal Medicine, the Federation of Asia and Oceania Perinatal Societies, the International Society of Perinatal Obstetricians. 2016;29(13):2205-9.
132. Wen L, Ge H, Qiao J, Zhang L, Chen X, Kilby MD, et al. Maternal dietary patterns and risk of gestational diabetes mellitus in twin pregnancies: a longitudinal twin pregnancies birth cohort study. Nutrition journal. 2020;19(1):13.
133. Wu K, Ke H-H, Gong W, Hu H, Chen L. Impact of Pre-Pregnancy Hemoglobin Level on the Association Between Pre-Pregnancy Body Mass Index and Gestational Diabetes Mellitus: A Retrospective Cohort Study in a Single Center in China. Diabetes, metabolic syndrome and obesity : targets and therapy. 2022;15(101515585):3767-75.
134. Xintong L, Dongmei X, Li Z, Ruimin C, Yide H, Lingling C, et al. Correlation of body composition in early pregnancy on gestational diabetes mellitus under different body weights before pregnancy. Frontiers in endocrinology. 2022;13(101555782):916883.
135. Xiong Y, Wang J, Qi Y, Liu C, Li M, Yao G, et al. Dose-response association between maternal pre-pregnancy bodyweight and gestational diabetes mellitus following ART treatment: a population-based cohort study. Reproductive biology and endocrinology : RB&E. 2022;20(1):92.
136. Yang J, Qian J, Qu Y, Zhan Y, Yue H, Ma H, et al. Pre-pregnancy body mass index and risk of maternal or infant complications with gestational diabetes mellitus as a mediator: A multicenter, longitudinal cohort study in China. Diabetes research and clinical practice. 2023;198(ebi, 8508335):110619.
137. Yao R, Park B, Browne K, Caughey A. Racial/ethnic differences in the association between obesity and gestational diabetes. American Journal of Obstetrics and Gynecology. 2017;216(1 Supplement 1):S306-S7.
138. Zaballa K, Liu A, Peek MJ, Mongelli M, Nanan R. Association between World Health Organization categories of body mass index and relative risks for weight-related pregnancy outcomes: a retrospective cohort study. Obstetric medicine. 2012;5(3):112-8.
139. Zhang J, An W, Lin L. The Association of Prepregnancy Body Mass Index with Pregnancy Outcomes in Chinese Women. Journal of diabetes research. 2022;2022(101605237):8946971.
140. Zhang R-Y, Wang L, Zhou W, Zhong Q-M, Tong C, Zhang T, et al. Measuring maternal body composition by biomedical impedance can predict risk for gestational diabetes mellitus: a retrospective study among 22,223 women. The journal of maternal-fetal & neonatal medicine : the official journal of the European Association

of Perinatal Medicine, the Federation of Asia and Oceania Perinatal Societies, the International Society of Perinatal Obstetricians. 2022;35(14):2695-702.
141. Zhao X, Lan Y, Shao H, Peng L, Chen R, Yu H, Hua Y. Associations between prepregnancy body mass index, gestational weight gain, and pregnancy outcomes in women with twin pregnancies: A five-year prospective study. Birth (Berkeley, Calif). 2022;49(4):741-8.
142. Zhao X, Li N, Jia R, Chen S, Wang L. The factors affecting the physical development of neonates in pregnant women with or without gestational diabetes mellitus. PloS one. 2021;16(4):e0251024.
143. Zheng Q-X, Wang H-W, Jiang X-M, Lin Y, Liu G-H, Pan M, et al. Prepregnancy body mass index and gestational weight gain are associated with maternal and infant adverse outcomes in Chinese women with gestational diabetes. Scientific reports. 2022;12(1):2749.
144. Zhou Z, Chen G, Li P, Rao J, Wang L, Yu D, et al. Prospective association of metal levels with gestational diabetes mellitus and glucose: A retrospective cohort study from South China. Ecotoxicology and environmental safety. 2021;210(edk, 7805381):111854.
145. Zonana-Nacach A, Baldenebro-Preciado R, Ruiz-Dorado MA. Effects of maternal and neonatal gestational weight gain. Salud Publica de Mexico. 2010;52(3):220-5.
